# Imperfection‐Enabled Strengthening of Ultra‐Lightweight Lattice Materials

**DOI:** 10.1002/advs.202402727

**Published:** 2024-09-16

**Authors:** Junhao Ding, Qingping Ma, Xinwei Li, Lei Zhang, Hang Yang, Shuo Qu, Michael Yu Wang, Wei Zhai, Huajian Gao, Xu Song

**Affiliations:** ^1^ Department of Mechanical and Automation Engineering Chinese University of Hong Kong Sha Tin Hong Kong 999077 China; ^2^ Faculty of Science, Agriculture, and Engineering Newcastle University Singapore 567739 Singapore; ^3^ Meta Robotics Institute Shanghai Jiao Tong University Shanghai 200240 China; ^4^ State Key Laboratory of Mechanical System and Vibration Shanghai Jiao Tong University Shanghai 200240 China; ^5^ Department of Mechanical Engineering National University of Singapore Singapore 117575 Singapore; ^6^ School of Engineering Great Bay University Songshan Lake Dongguan Guangdong 523808 China; ^7^ Mechano‐X Institute Applied Mechanics Laboratory Department of Engineering Mechanics Tsinghua University Beijing 100084 China

**Keywords:** geometric imperfections, strengthening effect, ultra‐lightweight lattice materials, yielding‐to‐buckling failure mode transition

## Abstract

Lattice materials are an emerging family of advanced engineering materials with unique advantages for lightweight applications. However, the mechanical behaviors of lattice materials at ultra‐low relative densities are still not well understood, and this severely limits their lightweighting potential. Here, a high‐precision micro‐laser powder bed fusion technique is dveloped that enables the fabrication of metallic lattices with a relative density range much wider than existing studies. This technique allows to confirm that cubic lattices in compression undergo a yielding‐to‐buckling failure mode transition at low relative densities, and this transition fundamentally changes the usual strength ranking from plate > shell > truss at high relative densities to shell > plate > truss or shell > truss > plate at low relative densities. More importantly, it is shown that increasing bending energy ratio in the lattice through imperfections such as slightly‐corrugated geometries can significantly enhance the stability and strength of lattice materials at ultra‐low relative densities. This counterintuitive result suggests a new way for designing ultra‐lightweight lattice materials at ultra‐low relative densities.

## Graphical abstract

This study identifies different compressive failure modes of cubic lattices with different relative densities and proposes a novel imperfection‐enabled strengthening mechanism of ultra‐lightweight lattice materials. Geometric imperfections are proven to be advantageous in enhancing the stability and strength of lattice materials at ultra‐low relative densities, which suggests a new way to strengthen ultra‐lightweight lattices via introducing imperfections for buckling prevention.

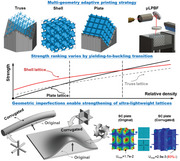

## 1 Introduction

Lightweight materials, characterized by low densities and superior stiffness and strength per unit mass, are crucial for modern engineering applications that demand increased material efficiency and fuel savings.^[^ ^1^, ^2^ ^]^ At present, these materials are employed in diverse fields including medical, automotive, and aerospace engineering.^[^ ^3^, ^4^ ^]^ One effective approach for weight reduction is incorporating various topological porosities into the materials.^[^ ^5^ ^]^ Lattice materials, which exhibit homogenized macroscopic properties, are a class of periodic microarchitectures resulting from this approach. They are designed to address the trade‐off between decreased relative density and the disproportionate drop in stiffness and strength.^[^ ^6^ ^]^ By tailoring internal microarchitectures, lattice materials can achieve a wide range of stiffness and strength,^[^ ^7^, ^8^, ^9^, ^10^ ^]^ as well as energy absorption capacity.^[^ ^11^ ^]^ The “stretching‐dominated” design concept is commonly used as a guideline for lattice materials in load‐bearing applications,^[^ ^12^ ^]^ aiming to reach the Hashin‐Shtrikman (HS)^[^ ^13^ ^]^ and Suquet^[^ ^14^ ^]^ upper bounds, representing the theoretical upper limits of stiffness and strength for isotropic elastic solids.

**Figure 1:**
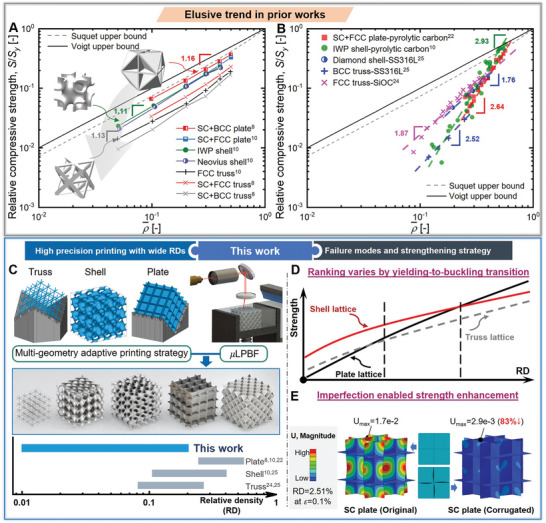
Schematic illustration of numerical and experimental results on the relative compressive strength of lattice materials from prior studies, and the methodologies and outcomes in this work. Comparison of the general trends of A) numerically evaluated,^[^ ^8^, ^10^ ^]^ and B) experimentally measured relative compressive strength, that is, the ratio of the compressive strength of the lattice (*S*) to the yield strength (*S_y_*) of the constitutive material, versus RD ($\bar{\rho }$) plots from prior studies.^[^ ^10^, ^22^, ^24^, ^25^ ^]^ C) The high‐precision µLPBF technique is adopted in this work to fabricate plate, shell, and truss lattice samples with a wide range of RDs (1.0–20.0%) for experimental investigation.^[^ ^8^, ^10^, ^22^, ^24^, ^25^ ^]^ D) Our numerical and experimental findings show that the winner of plate, shell, and truss lattices in strength varies with the RD due to the yielding‐to‐buckling failure mode transition. E) Introducing geometric imperfections such as slightly‐corrugated geometries into plate lattices enables buckling prevention, stability improvement, and strength enhancement at ultra‐low RDs.

Over the past decades, a variety of truss lattices, consisting of periodic networks of bars, have been designed to achieve superior stiffness and strength.^[^ ^15^, ^16^ ^]^ However, 1D bars can only exploit material loading in one direction. For stretching‐dominated truss lattices, the stiffness and strength are far below HS and Suquet upper bounds.^[^ ^17^, ^18^ ^]^ To achieve more exceptional load‐bearing efficiency, another class of lattice materials – shell lattices, which comprise periodic, open‐cell, smooth, and non‐intersecting shells, were developed,^[^ ^19^, ^20^ ^]^ exploiting more complex load‐bearing patterns beyond one direction. Due to their curved geometries, shell lattices generally bear external loads through a combination of in‐plane tension/compression and out‐of‐plane bending, often significantly outperforming truss lattices in stiffness and strength. However, bending effects can undermine the structural efficiency of shell lattices, making it still hard for them to reach the theoretical upper bounds.^[^ ^20^, ^21^ ^]^ Recently, stretching‐dominated plate lattices, which consist of periodic closed‐cell networks of plates, were proposed.^[^ ^7^, ^8^ ^]^ The constituent plates can transfer external loads into uniformly distributed stress within the tangent planes with negligible flexures. Therefore, in theory, plate lattices can be appropriately devised to approach the theoretical upper bounds at low relative densities (RDs),^[^ ^7^, ^8^, ^22^ ^]^ thus exhibiting optimal mechanical efficiency. Overall, the relevant studies reveal a trend of plate > shell > truss in strength for cubic lattices through numerical simulations,^[^ ^8^, ^10^ ^]^ as shown in the relative strength versus RD plot (**Figure** **1**A). However, the experimentally measured relative strength substantially decreases with decreasing RDs,^[^ ^10^, ^19^, ^22^, ^23^, ^24^, ^25^ ^]^ and the strength of isotropic plate lattices deviates even more from theoretical upper bounds at ultra‐low RDs,^[^ ^7^, ^8^ ^]^ exhibiting an inconsistent trend with numerical simulations (Figure 1B). These discrepancies highlight the necessity for a more in‐depth understanding of the mechanical behaviors of ultra‐lightweight lattice materials.

So far, different failure modes of lattice materials under compression, including material yielding and structure buckling, have been identified.^[^ ^26^, ^27^ ^]^ The buckling of microarchitectures tends to occur before yielding of constitutive materials at ultra‐low RDs, leading to a significant reduction in relative strength.^[^ ^28^, ^29^ ^]^ The yielding‐to‐buckling failure mode transition has been sparsely observed in limited prior studies.^[^ ^29^, ^30^, ^31^ ^]^ However, these studies did not systematically summarize it, nor incorporate this transition into numerical simulations, let alone integrating it as a part of design considerations.

In this work, we fully elucidate the yielding‐to‐buckling failure mode transition during compression in cubic lattices and propose a novel strategy to strengthen ultra‐lightweight lattice materials by introducing imperfections for buckling prevention. Herein, three classes of cubic lattices, namely plate, shell, and truss lattices, with a wide range of RDs (1.0–20.0%) are fabricated via an advanced high‐precision micro‐laser powder bed fusion (µLPBF) technique (Figure 1C), based on a well‐designed multi‐geometry adaptive printing strategy. This strategy enables to maintain homogeneous mechanical properties of constitutive materials and ensure high manufacturing fidelity. Combined with numerical simulations, the different types of deformation behaviors and the yielding‐to‐buckling failure mode transition under compression are well captured. Notably, the “stretching‐dominated” concept‐enabled ranking in strength, that is, plate > shell > truss, is only true at moderate RDs (i.e., >4.7%), when the dominant failure mode is material yielding. At ultra‐low RDs, shell and truss lattices significantly outperform plate lattices, resulting in a remarkably different ranking in strength (Figure 1D). The structural stability and mechanical efficiency are highly influenced by the bending strain energy ratio (BSER). By increasing BSERs through introducing imperfections such as slightly‐corrugated geometries into the lattice design, the strength of lattice materials can be significantly enhanced via buckling prevention at ultra‐low RDs (Figure 1E). Overall, our study provides valuable insights into the strength of lattice materials at ultra‐low RDs and delivers effective design guidelines to strengthen ultra‐lightweight lattice materials.

## 2 Results

### 2.1 High‐Precision Micro‐3D Printing and Yielding‐To‐Buckling Failure Mode Transition

**Figure 2:**
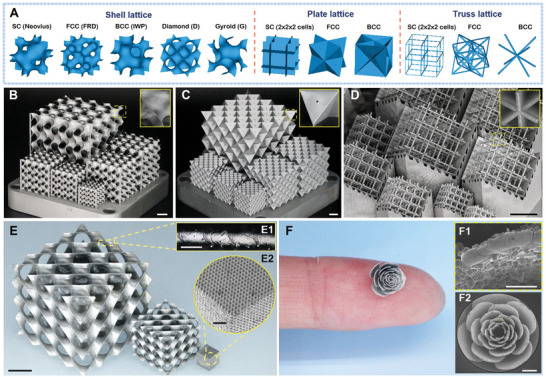
Three classes of cubic lattices and µLPBF fabricated metallic samples: A) Selected plate, shell, and truss lattices for numerical simulations, including Neovius, FCC rhombic dodecahedron (FRD), I‐graph‐wrapped package (IWP), D, G TPMS shell lattices, and SC, FCC, BCC plate and truss lattices. µLPBF fabricated metallic samples of B) G shell lattices, C) FCC plate lattices, D) SC truss lattices, and E) D shell lattices, for experimental investigation. F) Illustration of the fabrication accuracy of our high‐precision µLPBF technique via a tiny metallic flower sample. Scale bars are 10 mm in (B–E), 100 µm in (E1,F1), and 1 mm in (E2,F2), respectively.

Here, via the combination of numerical and experimental analysis, we investigate the compressive strength and failure modes of the three classes of cubic lattices (**Figure** **2**A). We confirm that cubic lattices under compression undergo a yielding‐to‐buckling failure mode transition, which is typically characterized by a significant reduction in strength. The transition RD and reduction amount in strength vary by the lattice type (Section S1, Supporting Information). Gyroid (G), Diamond (D) triply periodic minimal surface (TPMS) shell lattices (Figure 2B,E), simple cubic (SC), face‐centered cubic (FCC) plate lattices (Figure 2C), and SC truss lattices (Figure 2D) were selected for experimental tests due to their superior strength within the respective categories based on numerical post‐buckling analysis (Section S1, Supporting Information). The unit cell sizes and RDs of samples were within the range of 2.0–20.0 mm and 1.0–20.0%, respectively. The samples were fabricated via µLPBF, using stainless steel 316 L (SS316L) as the constitutive material (Section S2, Supporting Information). This ductile metallic material has a yield strength, *S_y_* ≈ 520 MPa, Poisson's ratio, *ν_s_* ≈ 0.29, Young's modulus, *E_s_* ≈ 190 GPa, and strain hardening modulus, *E_h_* ≈ 967 MPa. Its mechanical properties are insensitive to the feature size and scanning strategies within the range of interest in this work (Sections S2 and S3, Supporting Information). While plate, shell, and truss lattices face different manufacturing challenges, we confidently affirm the effectiveness of our multi‐geometry adaptive printing strategy by the high fidelity and high dimensional accuracy of the as‐printed samples. We have successfully fabricated ultra‐thin‐walled structures with a minimum thickness of 65 µm, and various complex geometries at the limit of machine resolution (Figure 2E,F; Section S3, Supporting Information). These manufacturing capabilities empower us to minimize the influence of various factors, including manufacturing defects, constitutive materials, and size effects. Thus, we can focus on the effects of microarchitecture geometries on the mechanical properties of lattice materials.

**Figure 3:**
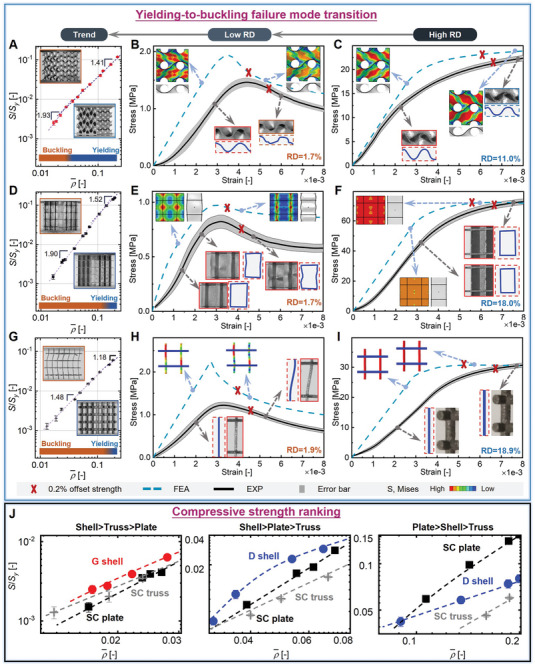
Experimentally measured and numerically evaluated effective compressive strength, stress–strain curves, and strength ranking of G shell, SC plate, and SC truss lattices with varying RDs. Experimentally measured relative strength versus RD plots of A) G shell lattices, D) SC plate lattices, and G) SC truss lattices. Numerically evaluated and experimentally measured stress–strain curves of B,C) G shell lattices, E,F) SC plate lattices, and H,I) SC truss lattices with different RDs. J) Experimentally measured strength ranking of G shell, SC plate, and SC truss lattices at different RD regimes.

We have identified the deformation behaviors, failure modes, and compressive strength of the three classes of lattices with various RDs. To further elaborate on this point, lattices under compressive loading may experience two primary types of failure modes: constitutive material yielding and lattice structure buckling. At sufficiently high RDs, all types of cubic lattices exhibit material yielding failure, without any evident structure buckling failure, which is referred as the yielding failure mode. As the RD decreases, the lattices are more prone to structure buckling failure during compression, which is referred as the buckling failure mode. A reduction in RD typically leads to a disproportionate decrease in strength, characterized by the scaling relationship $S\propto S_{y}{\bar{\rho }}^{n}$, where $\bar{\rho }$ is the RD, *S_y_* denotes the yield strength of the constitutive material, and the exponent *n* serves as a scaling factor dependent on the lattices. However, the occurrence of buckling failure generally results in a further reduced load‐bearing capacity and an expedited reduction in strength as the RD decreases further, thereby leading to a higher exponent *n* at lower RDs. Experimental results demonstrate that the relative strengths of G shell lattices experience two decreasing stages, corresponding to $S/S_{y}\propto {\bar{\rho }}^{1.41}$ ($\bar{\rho }>2.4\%$) and $S/S_{y}\propto {\bar{\rho }}^{1.93}$ ($\bar{\rho }<2.4\%$), respectively (**Figure** **3**A). Thus, a failure mode transition point of G shell lattices can be found at an RD ≈2.4%, which is estimated as the intersection point of the two fitted linear straight lines of the corresponding stages. The experimentally measured transition RD of 2.4% is well aligned with the numerically predicted RD range of 1.6–2.5% (Section S1, Supporting Information). Based on the different deformation behaviors of the lattices in this RD regime, this point is identified as the yielding‐to‐buckling transition point.

More specifically, the numerically evaluated and experimentally measured stress–strain curves of G shell lattices with two representative RDs (1.7% and 11.0%) are illustrated in Figure 3B,C, respectively. The lattice ($\bar{\rho }=1.7\%$) undergoes plastic buckling failure, wherein the stress–strain curve initially exhibits a linear growth until the initial yield point. Subsequently, the curve shows a nonlinear growth due to the yielding of constitutive materials, and then decreases significantly after reaching the peak stress, owing to the onset of plastic buckling behavior. The buckling phenomenon is also manifested by the morphological change of shell edges, which undergo significant distortions upon reaching the initial peak stress (Figure 3B). In contrast, the lattice ($\bar{\rho }=11.0\%$) undergoes material yielding failure, wherein the stress–strain curve first increases linearly until the initial yield point, and then increases nonlinearly until the 0.2% offset yield point. The sample edges keep original shapes upon yielding of constitutive materials, indicating no occurrence of buckling behaviors (Figure 3C).

In comparison, the buckling failure of plate and truss lattices occurs at an earlier stage than shell lattices. SC plate lattices undergo the yielding‐to‐buckling failure mode transition at RDs near 10.5%, matching well with the numerically predicted range of 10.0–15.0%. The slope of the relative strength versus RD curve increases from 1.52 to 1.90 (Figure 3D). The numerical results indicate that SC plate lattice ($\bar{\rho }=1.7\%$) undergoes elastic buckling failure. This failure mode is characterized by a stress–strain curve that initially exhibits a linear growth until the critical buckling strength. Subsequently, the curve shows a nonlinear growth until a peak stress, and then decreases due to the yielding of constitutive materials. The buckling behavior is also validated by the deformation patterns of the lattice, wherein vertical plates are curved and bulge out under external loads (Figure 3E). In contrast, the lattice ($\bar{\rho }=18.0\%$) undergoes material yielding failure, wherein vertical plates remain straight upon yielding of constitutive materials (Figure 3F). The experimental results show a consistent trend with numerical ones and validate different deformation behaviors of SC plate lattices at the two different RDs. Both material yielding and elastic buckling result in a decrease of the slope of the stress–strain curve, while elastic buckling typically results in a larger slope reduction than material yielding. Besides, plastic buckling generally induces a more significant decrease of the stress–strain curve after the peak stress than material yielding. Moreover, SC truss lattices are found to undergo the yielding‐to‐buckling transition at RDs near 12.1%, with the slope of the relative strength versus RD curve increased from 1.18 to 1.48 (Figure 3G). The lattice ($\bar{\rho }=1.9\%$) undergoes material yielding and structure buckling failures almost simultaneously, with a sudden drop of the stress–strain curve upon reaching the first peak stress (Figure 3H). The material yielding failure occurs at a higher RD ($\bar{\rho }=18.9\%$), wherein vertical bars remain almost straight under external loads (Figure 3I). Comparatively, G shell lattices have the lowest transition RD and a moderate reduction in strength, thereby demonstrating the best lightweight potential in strength as compared to other examined lattices. The remaining lattices exhibit similar deformation behaviors and failure mode transition, and the detailed results are illustrated in Section S1 (Supporting Information).

Overall, the strength of the three classes of lattices is ranked as plate > shell > truss, shell > plate > truss, and shell > truss > plate at high, intermediate, and ultra‐low RDs (Figure 3J), respectively. The three RD intervals are split according to the different failure modes of lattices (i.e., material yielding, plastic buckling, and elastic buckling). Although their specific values may vary depending on the lattice type, the fundamental principle of this division remains the same. The widely perceived trend of plate > shell > truss is only true at RDs above 4.7% (Section S1 and Figure S1d,d‐1, Supporting Information), when the predominant failure mode of most lattices is material yielding. The stretching‐dominated design principle works well in this RD regime, and the lattice with higher mechanical efficiency exhibits higher compressive strength. However, plate lattices undergo the yielding‐to‐buckling failure mode transition earlier than shell lattices, therefore the ranking gradually changes into shell > plate > truss at 1.6–4.7% RDs (Section S1 and Figure S1d,d‐2, Supporting Information). As the RD further decreases below 1.6%, the ranking becomes shell > truss > plate (Section S1 and Figure S1d,d‐3, Supporting Information), wherein the strength of lattices at such low RDs is dominated by buckling of microarchitectures. Notably, G shell lattice with nearly isotropic elasticity, demonstrates the highest strength at ultra‐low RDs compared with other examined lattices. This superior strength is primarily attributed to the lower yielding‐to‐buckling failure mode transition RD of G shell lattices, and the moderate reduction in their strength with decreasing RDs. Comparatively, SC plate and truss lattices undergo the yielding‐to‐buckling transition at higher RDs, with a more considerable reduction in strength with decreasing RDs, thus exhibiting lower strength than G shell lattices at ultra‐low RDs. Furthermore, SC plate and truss lattices also exhibit highly anisotropic elasticity, with [100] being the stiffest direction, which further demonstrates the exceptional strength of G shell lattices along different directions (Section S4, Supporting Information).

### 2.2 Strain Energy Analysis and Imperfection‐Enabled Strength Enhancement at Ultra‐Low RDs

To elucidate the underlying failure mechanisms among the three classes of lattices, we investigate the distribution, evolution, and composition of strain energy under compressive loading within a strain range from 0% to 1%. The strain energy distributions within lattice microarchitectures greatly affect their mechanical performances, and the deformation behavior under finite strain is of utmost importance as it dictates the reaction to external loads.

**Figure 4:**
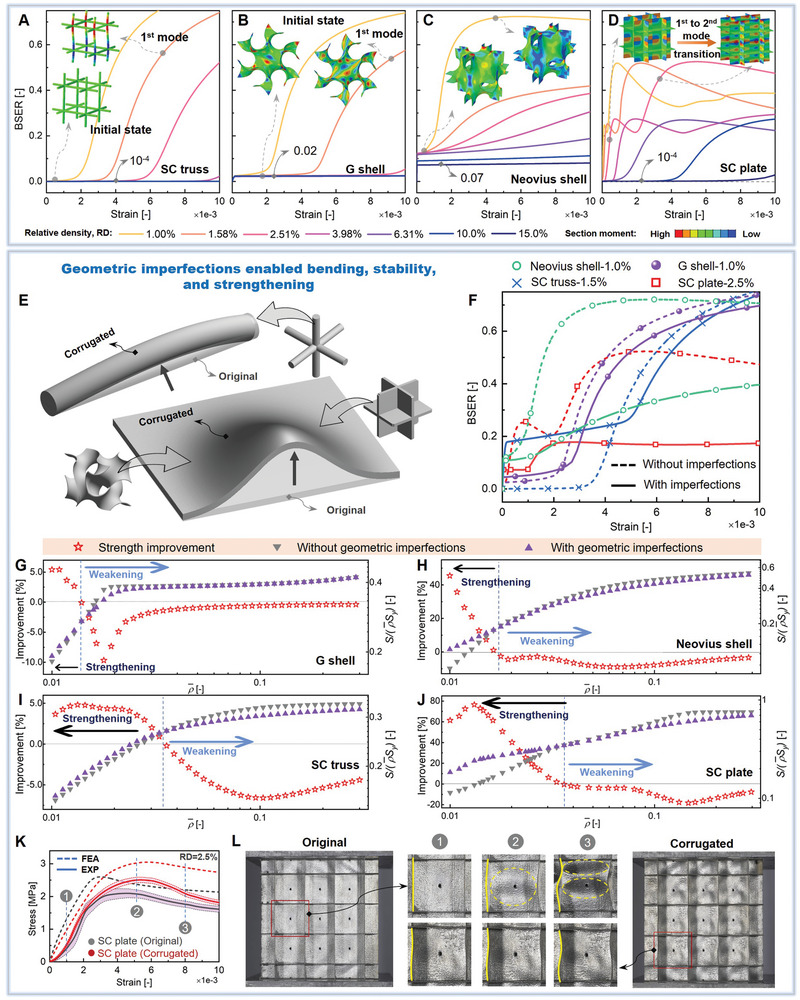
Summary of the strain energy analysis and imperfection‐enabled strengthening effect of ultra‐lightweight lattice materials: the BSERs of A) SC truss, B) G shell, C) Neovius shell, and D) SC plate lattices with different RDs, the schematic illustration of E) geometric imperfections and F) the BSER curves of lattices with/without imperfections, the effect of imperfections on the compressive strength of G) G shell, H) Neovius shell, I) SC truss, and J) SC plate lattices, and K,L) experimental validations.

Here, we introduce a useful parameter/indicator: BSER, the ratio of bending strain energy to total strain energy (Section S5, Supporting Information), to probe the deformation behavior of lattices and quantify the effects of deformation modes (bending versus stretching, stability versus buckling) on their mechanical performances. At high RDs, the BSERs of SC plate, FCC plate, and SC truss are ≈10^−4^ due to their nearly pure stretching deformations, while the BSERs of G, Neovius, and D shells are 0.02, 0.07, and 0.005, respectively (**Figure** **4**A–D; Figure S14a,b, Supporting Information). In low RD regimes, all types of lattices exhibit rising BSERs under increasing compressive strains. Notably, SC plate and Neovius shell experience a remarkable rising in BSER at the early stage of compression in extremely low strains, whereas G shell and SC truss exhibit a relatively stable stage of low BSERs, succeeded by a gradual increase. The BSER curve of SC plate shows a multi‐stage pattern, characterized by an initial increase, then a minor reduction and a subsequent secondary increase. The two stages of increase are attributed to the transition between the first‐order and second‐order buckling modes, as depicted in the sectional moment resultants *SM*_2_ (Figures 3E and 4D). In contrast, the G shell, Neovius shell, and SC truss only exhibit the first‐order buckling mode. The observed disparity can be predominantly attributed to the distinct buckling modes inherent in various types of lattices. The first‐order and second‐order buckling modes of SC plate lattices exhibit a comparable level of buckling strength and deformation patterns, while other lattices display significantly different deformation patterns that are unlikely to occur concurrently or progressively (Section S5, Supporting Information). During the buckling and post‐buckling stages, the stress and strain energy typically experience a redistribution, thus leading to a variation of the BSER. Indeed, the buckling behavior is a mechanism with a rapid transition to quickly redistribute the strain energy to attain a new state of stability. The transition mechanism is also validated by the cumulative distribution of strain energy density. SC plate and truss exhibit a characteristic step function distribution, which then undergoes a fast transition toward a smoother profile (Section S6, Supporting Information). In case of stretching‐dominated lattices, the occurrence of buckling induces flexural deformations, causing a significant rise in BSER, thereby compromising the load‐bearing capacity. Our investigation reveals that the compressive strengths of lattice materials are related to both the initial value and progression of BSER. Notably, SC plate lattices, with the lowest (near zero) initial value of BSER, exhibit the highest strength at high RDs. In contrast, D shell lattices with slightly larger BSERs surpass SC plate lattices and demonstrate the highest strength at intermediate RDs, since SC plate lattices exhibit increased BSERs in this RD regime.

Owing to the yielding‐to‐buckling failure mode transition, a different design strategy can be employed to strengthen ultra‐lightweight lattices via buckling prevention. In this study, we demonstrate that imperfections, such as slightly‐corrugated geometries (Figure 4E; Section S7, Supporting Information), can play a crucial role in enhancing the stability of stretching‐dominated lattices at ultra‐low RDs. This approach is effectively demonstrated by implementing a minor periodic offset of the middle plane/line to create slight corrugations. Specifically, given the curved nature of shell lattices, the imperfections were intentionally introduced in regions with low net curvatures (Section S7, Supporting Information). The findings demonstrate that the implementation of slightly‐corrugated geometries can improve the initial bending strain energy of the three classes of lattices (Figure 4F). This improvement enhances the stability of lattices, as validated by their BSER curves. Particularly, the imperfect surface brings the BSER of SC plate lattice from 10^−4^ to ≈0.07 at the initial state. During the compression, the BSER increases and stabilizes at ≈0.17. Furthermore, the importance of initial BSER on the structural stability of lattices is validated by evaluating the sensitivity of strain energy on the random geometric noise (Section S7 and Figure S18, Supporting Information). We find that SC plate has a high susceptibility to geometric noise, and the introduction of noise leads to a significant reduction in strain energy. The stability of SC plate was improved by introducing initial bending effects to augment the initial BSER, wherein the introduction of random geometric noise only induces a slight reduction in strain energy. Comparatively, G shell has a notable degree of inherent stability, whose strain energy is almost unaffected by the geometric noise.

The effect of imperfections on the compressive strength depends on the RD and type of lattices (Figure 4G–J). Imperfections can strengthen all three classes of lattices at ultra‐low RDs via improving the buckling strength, albeit the degree of enhancement varies with the lattice type. In case of high RDs with greater structural stability, which is unlikely to buckle, further increases in critical buckling strength do not contribute to an improved strength. Notably, lattices with higher inherent stability, such as SC truss and G shell, exhibit lower enhancements through the introduction of imperfections, which, however, turns into the weakening effect at high RDs. In contrast, Neovius shell and SC plate lattices exhibit lower inherent stability, thus demonstrating substantial enhancement in strength as a result of the incorporation of imperfections. Specifically, SC plate lattices exhibit three distinct decreasing stages, each of which correspond to a specific failure mode. As the RD decreases, SC plate lattices experience material yielding, plastic buckling, and elastic buckling failures successively, with transition RDs occurring at ≈13.5% and 3.6%, respectively. Comparatively, the introduction of corrugations delays the onset of elastic buckling to an RD near 1.3%, and results in an enhanced strength at RDs below 3.6% (Figure 4J). Compared with material yielding failure, structure buckling failure usually results in a more significant reduction in relative strength as the RD decreases. Therefore, the strength can be enhanced by inhibiting buckling at low RD regimes, where structure buckling is the dominant failure mode.

In this study, SC plate lattices are selected as a representative case for the experimental validation of the imperfection‐enabled strengthening mechanism. Through numerical simulations, the incorporation of corrugations into SC plate lattices is found to improve the buckling strength by 81% and enhance the overall compressive strength by ≈20% at 2.5% RD (Figure 4K). According to experimental results, the first peak stress of the original lattice occurred at a lower strain of 0.45%, whereas the corrugated lattice reached the first peak stress at a higher strain of 0.56% (Figure 4K; Movie S1, Supporting Information). Importantly, the corrugated lattice maintained its original shape, with no discernible changes in structural configurations, demonstrating enhanced stability. In comparison, the original lattice exhibited evident signs of buckling failure at a lower strain of 0.3%, as illustrated by its curved edges and invaginated planes (Figure 4L; Movie S1, Supporting Information). In comparison, the enhanced stability of the corrugated lattice resulted in increased strength.

Furthermore, at the large strain ranges, the deformation behavior of the corrugated lattice followed a layer‐by‐layer collapse pattern, where the deformation was primarily confined to a single layer, while other layers maintained their original configurations. In contrast, the original lattice exhibited a non‐uniform deformation pattern. Notably, each peak stress observed in the corrugated lattice, representing the collapse of a layer, was higher than that of the original lattice (Movie S2, Supporting Information). Therefore, the corrugated lattice also exhibited higher stability and enhanced strength at large strain ranges.

To summarize, imperfections are demonstrated to substantially strengthen lattice materials at ultra‐low RDs via stability enhancement, in which structure buckling takes over as the dominant failure mode. In contrast, imperfections generally decrease the strength of lattice materials at moderate or high RDs, in which the dominant failure mode of most lattices is material yielding.

### 2.3 Comparison with Prior Studies

**Figure 5:**
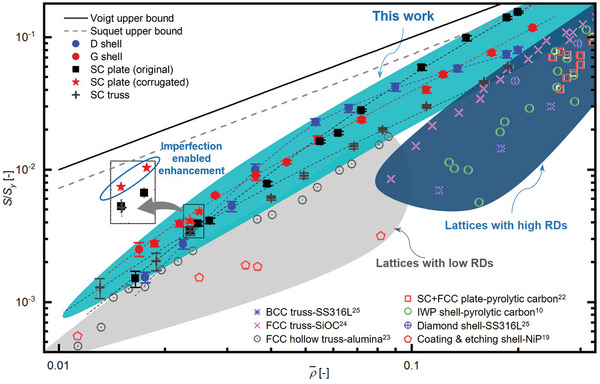
Comparison of the experimentally measured relative strength of lattice samples in this work to those in prior studies, the Voigt upper bound, and Suquet upper bound.^[^ ^10^, ^19^, ^22^, ^23^, ^24^, ^25^ ^]^

The experimentally measured relative strengths of lattice samples are further compared with those in prior studies,^[^ ^10^, ^19^, ^22^, ^23^, ^24^, ^25^ ^]^ the Voigt upper bound,^[^ ^10^ ^]^ and Suquet upper bound (**Figure** **5**).^[^ ^14^ ^]^ Generally, our high‐precision µLPBF technique enables the fabrication of lattice samples with a much wider range of RDs than most prior studies, especially in the RD range below 10.0%. Due to the superior accuracy and quality of our µLPBF technique, the samples in this work exhibit superior relative strength to those in prior studies. Specifically, SC plate lattices approach the Suquet upper bound of yield strength above 10.0% RDs. The µLPBF fabricated metallic lattices also outperform their counterparts of micro/nano lattices with equivalent RDs.^[^ ^19^, ^23^ ^]^ The G shell lattices in this work possess similar power‐law scaling relation and exhibit higher relative strength than those in prior studies at 1.0–8.0% RDs. While some prior studies lack experimental data below RDs of 10.0%, it can be inferred from the samples analyzed in this study that our samples also demonstrate much superior relative strength below 10.0% RDs. This inference is drawn from the observation that the relative strength of samples in prior studies experiences a more substantial decrease at RDs close to 10.0% (Figure 5).

## 3 Further Discussion

To comprehensively unveil the lightweight capabilities of metallic lattice materials, the precise fabrication of metallic lattices encompassing a broad spectrum of RDs is imperative. Additionally, the formulation of a design guideline specifically tailored for ultra‐low RDs is required. These tasks are by no means inconsequential, as the yielding of constitutive materials and buckling of microarchitectures can manifest either independently or concurrently, and their interactions can substantially affect the mechanical properties of lattice materials. Combining numerical post‐buckling analysis and experimental tests, this work distinguishes different failure modes of lattice materials with varying RDs, including elastic buckling, plastic buckling, and material yielding. The lattices are fabricated using SS316L as the constitutive material, whose mechanical properties are stable and insensitive to the laser scanning parameters within the range of interest (Sections S1 and S2, Supporting Information). Together with our multi‐geometry adaptive printing strategy and high‐precision µLPBF system, the RD range of our samples is expanded to a wider range (from 1.0% to 20.0%) than other manufacturing techniques while maintaining high fidelity. Accordingly, different failure modes are experimentally captured using identical materials processed by the same fabrication technique. In this study, the RD was selected as an influencing factor to evaluate the strength of lattices. As an alternative, the aspect ratio can also be adopted as an indicator throughout the evaluation process. In practice, the two factors are geometric descriptions of the same lattices from two different perspectives (Section S8, Supporting Information).

Our work indicates that the type of strength‐optimal lattices varies with RD, and a dominant winner no longer exists. The findings underscore the limitations associated with stretching‐dominated design concept. Specifically, the assumption of negligible flexural deformations under low RDs is overly simplistic. For given constitutive materials, the strength of lattice materials is governed by both mechanical efficiency and structural stability, and the significance of structural stability is crucial for lattices with ultra‐low RDs. The primary benefits of plate lattices are derived from their constituent plates, which exhibit a nearly pure membrane stress state.^[^ ^7^, ^8^ ^]^ However, the occurrence of buckling behavior becomes unavoidable in ultra‐low RD regimes, resulting in a significant rise in BSER and a substantial reduction in load‐bearing capacity. These findings are also applicable to other constitutive materials, including those with higher yield strength to Young's modulus ratios, *S_y_/E_s_*, such as pyrolytic carbon,^[^ ^22^ ^]^ ceramic,^[^ ^23^, ^24^ ^]^ etc. For those materials, the buckling‐induced strength reduction will be more pronounced and result in an earlier yielding‐to‐buckling failure mode transition at higher RDs.^[^ ^26^ ^]^ For instance, pyrolytic carbon plate lattices exhibit a significant reduction in relative strength with a high scaling factor and are surpassed by octet truss lattices at an RD near 25.0%.^[^ ^22^ ^]^

Hence, beyond the stretching‐dominated design concept, it is imperative to redirect our attention toward enhancing structural stability while simultaneously maintaining optimal mechanical efficiency at ultra‐low RDs. Our investigation on strain energy provides a clear elucidation of the underlying mechanisms that govern the yielding‐to‐buckling failure mode transition. Generally, buckling behaviors tend to redistribute the stress and strain energy of lattices with low stability to transit into a state of greater stability. Geometric imperfections are typically perceived as undesirable factors due to their propensity to induce stress concentration and flexural deformation. However, a distinct phenomenon emerges when considering imperfections within ultra‐low RD regime. We have observed a compelling association between the BSER curve and the overall structural stability of lattice materials. Surprisingly, artificially implemented imperfections that introduce appropriate bending effects are proven to be advantageous in guaranteeing the structural stability and enhancing the strength at ultra‐low RDs. This insight challenges the conventional understanding of imperfections as solely detrimental to lattice materials. By acknowledging the potential positive effect of imperfections at ultra‐low RDs, we can explore new avenues for optimizing lattice designs and exploiting imperfections to enhance their structural performances.

## 4 Conclusion

In summary, our study presents a full map of different failure modes in cubic lattices across a broad range of RDs and, in doing so, unveils a novel imperfection‐enabled strengthening mechanism for ultra‐lightweight lattice materials. The manufacturing challenges inherent in the three distinct classes of metallic lattices with ultra‐low RDs are effectively overcome by the integration of high‐precision micro‐LPBF and a multi‐geometry adaptive printing approach. Through quasi‐static compression tests and advanced numerical simulations, our study successfully captures the failure mode transition from material yielding to structure buckling, leading to a fundamental re‐evaluation of the conventional wisdom in the strength ranking of the three classes of lattices. The usual strength ranking of plate > shell > truss only holds true at high RDs, while the ranking gradually changes to shell > plate > truss or shell > truss > plate at low RDs. Particularly, the introduction of a novel metric term BSER in the design process sheds light on the underlying mechanisms governing the failure mode transition. Our results demonstrate that introducing appropriately tailored geometric imperfections into lattice materials can provide significant advantages to enhance their strength at ultra‐low RDs. This counter‐intuitive observation presents a new approach to strengthen ultra‐lightweight lattice materials via buckling prevention.

## 5 Experimental Section

### Numerical Method

Numerical simulations of lattices with varying RDs (1.0–20.0%) were implemented using the commercial ABAQUS 2022 software. First, the linear static analysis and linear eigenvalue buckling analysis were performed to determine the stiffness and first‐order elastic buckling mode of the lattices, respectively. Subsequently, the nonlinear static analysis was performed to obtain the stress–strain curves and 0.2% offset strength.^[^ ^8^, ^10^, ^22^ ^]^ The first‐order elastic buckling mode was multiplied by different factors and incorporated into the analysis as initial deformation behaviors, which serves as a convergence study on the multiplication factors.^[^ ^32^ ^]^ A representative volume element (RVE) of the lattices was selected for analysis, and periodic boundary conditions were imposed in all three directions. SC plate/truss lattices consist of three mutually perpendicular plates/bars within the unit cell, and the boundary edges of their unit cells are simply, continuously, and smoothly connected to the neighboring cells, therefore the trans‐cell buckling modes cannot be restricted. The first‐order elastic buckling wavelength of SC plate lattices was demonstrated to be twice of the unit cell size,^[^ ^32^ ^]^ and the 2 × 2 × 2 periodic array of unit cells was shown to capture the most common buckling modes of SC truss lattices.^[^ ^33^ ^]^ Therefore, the 2 × 2 × 2 unit cells were adopted as the RVE for numerical analysis of SC plate/truss lattices in this work. In contrast, the unit cell of the remaining lattices was selected as the RVE for analysis, since their first‐order elastic buckling modes occurred within the unit cell. The S3 shell element, a Reissner‐Mindlin plate/shell theory‐based three‐node triangular shell element, was adopted for analysis of plate and shell lattices, with a total element number between 15 000 and 35 000. The B31 beam element, a Timoshenko‐Ehrenfest beam theory‐based two‐node linear beam element, was adopted for analysis of truss lattices, with a total element number between 2500 and 5500. The manufacturing defects, obtained from micro‐CT reconstructed models (Section S3, Supporting Information), were also incorporated into the simulations. The mechanical properties of the constitutive material, SS316L, were obtained through uniaxial tensile tests of the µLPBF fabricated standard dogbone samples (Section S3, Supporting Information).

### Geometric Modeling and Toolpath Generation

The TPMS mid‐surfaces of shell models were constructed via an open‐source software, Surface Evolver.^[^ ^34^ ^]^ Then, various shell lattices with different cell sizes and thicknesses were generated in MATLAB via rescaling and offset of the stereolithography (STL) mesh files. Plate and truss lattices were modeled in the software package SolidWorks 2020. The removal of powders was made possible by introducing circular holes with 400 µm diameter in the center of plate mid‐surfaces. Multiple studies have shown that these small holes do not substantially affect the mechanical properties of the lattices.^[^ ^8^, ^22^ ^]^ Low RDs were achieved via scaling up the unit cell size while keeping constant wall thicknesses of plate/shell lattices or cross‐sectional diameters of truss lattices. All geometric models were exported as STL files for slicing, and then the laser scanning path was generated according to the sliced contours via an in‐house developed MATLAB algorithm (Section S2, Supporting Information).

### Fabrication Method

Various metallic lattices were printed by an in‐house developed high‐precision µLPBF platform, Hans M100 µ, fitted with an Yb laser source (*λ* = 1.07 µm) with a beam spot size of 25 µm (Figure S2, Supporting Information). Austenitic stainless steel SS316L with the powder size distribution of 5‐25 µm (*D*_50_ = 16.27 µm) was selected for printing. Considering the building volume, the plate, truss, and D shell lattices with 4 × 4 × 4 unit cells were printed, while 3 × 3 × 3 arrangements for G shell lattices were adopted at low RDs. Two different scanning strategies were designed for different lattice materials, including the single track along the mid‐surfaces of the models and R67 path with contour scanning. Correspondingly, dogbone samples with the gauge dimensions of 10 × 2 × 0.07 mm^3^ and 20 × 4 × 1.5 mm^3^ were fabricated for constitutive material characterizations (Figure S4, Supporting Information). The slicing and toolpath generation were conducted via an in‐house developed MATLAB algorithm. To avoid the large overhang regions of SC plate and truss lattices, the lattices were rotated and printed along [110] and [111] directions, respectively (Figure S2, Supporting Information). After finishing printing, the samples and supports were cut off from the building plate via electrical discharge machining.

### Characterization Method

The RDs of samples were calculated based on their actual measured mass and dimensions. To prevent an overestimation of mass due to stuck particles, the RDs of SC and FCC plate lattices were measured as those of their unit cells (Figure S7, Supporting Information). Conversely, the RDs of the remaining lattices were measured on multi‐cell samples directly, since they were free from stuck particles due to the open‐cell property. The vertical wall thickness and surface quality were characterized via a 3D optical microscope, RH‐2000, HIROX. The morphologies of the lattice samples and metallic flowers were characterized by a scanning electron microscope (SEM, JCM‐6000Plus). The manufacturing fidelity along the 3D directions was characterized by micro‐CT, Scanco Medical µCT‐35 equipped with a 70 kV X‐ray source (Figure S8, Supporting Information). The wall thicknesses of plate and shell lattices, and the cross‐sectional diameters of truss lattices were analyzed via an in‐house developed algorithm based on the reconstructed models. Quasi‐static compression tests were carried out on an MTS machine (Model 370.1, MTS Landmark Testing Solutions). Specifically, the Young's moduli were calculated according to the unloading curves obtained by unload‐reload cyclical compression tests. The compressive strength was estimated by the 0.2% offset point of the engineering stress–strain curves obtained by uniaxial compression tests at a constant nominal strain rate of 0.001 s^−1^.^[^ ^8^, ^10^, ^22^ ^]^ The properties of constitutive materials were characterized by uniaxial tensile tests of the standard dogbone samples according to ASTM E8/E8M standard (Figure S4, Supporting Information). The repeatability of the experiment was validated using at least three repeated test samples, and the average and variation of mechanical responses were derived from these results (Section S3.4, Supporting Information).

## Conflict of Interest

The authors declare no conflict of interest.

## Supporting information

Supporting information

Supplemental Movie 1

Supplemental Movie 2

## Data Availability

The data that support the findings of this study are available from the corresponding authors upon reasonable request.
