# Supplementary material for: Imperfection‐Enabled Strengthening of Ultra‐Lightweight Lattice Materials
Source: Adv Sci (Weinh). 2024 Sep 16;11(41):2402727. doi: 10.1002/advs.202402727 (PMC11538692; doi:10.1002/advs.202402727)
Supplement: Supplementary file 1 — Supporting information [file ADVS-11-2402727-s003.docx]

Supporting Information

**Imperfection-Enabled Strengthening of Ultra-Lightweight Lattice Materials**

*Junhao Ding^†^, Qingping Ma^†^, Xinwei Li, Lei Zhang, Hang Yang, Shuo Qu, Michael Yu Wang, Wei Zhai^*^, Huajian Gao^*^, Xu Song^*^*

^†^ These authors contributed equally: Junhao Ding, Qingping Ma.

^*^ Corresponding authors: Wei Zhai (mpezwei@nus.edu.sg), Huajian Gao (gao.huajian@tsinghua.edu.cn), Xu Song (xsong@cuhk.edu.hk).

**1. Numerical post-buckling analysis**

We investigate the compressive strength and failure modes of the three classes of cubic lattices via numerical post-buckling analysis, including simple cubic (SC), face-centered cubic (FCC), and body-centered cubic (BCC) plate lattices, truss lattices, together with Neovius, FCC rhombic dodecahedron (FRD), I-graph-wrapped package (IWP), Diamond (D), and Gyroid (G) triply periodic minimal surface (TPMS) shell lattices. First, the strength of three representative lattices (including D shell, SC plate, and SC truss lattices) with varying relative densities (RDs) (1.0%-20.0%) is investigated through numerical post-buckling simulations. The numerical results reveal that incorporating initial buckling behaviors has little effect on the strength of D shell lattices (**Figure S1a**). In contrast, a significant reduction in strength is observed for SC plate and truss lattices, especially at low RDs when the critical buckling strength is lower than or comparable to yield strength (**Figure S1b** and **c**). The relative strength versus RD plot of D shell lattices shows a sudden drop at 2.5%-4.0% RDs. Besides, the lattices exhibit significantly different deformation patterns and von Mises stress distributions at the two RDs (Figure S1a). Therefore, D shell lattices are demonstrated to undergo the yielding-to-buckling failure mode transition at 2.5%-4.0% RDs. Similarly, SC plate and truss lattices are shown to undergo the yielding-to-buckling transition at higher RDs between 10.0% and 15.0% (Figure S1b and c).

Subsequently, the strength of all selected lattices is investigated using the same numerical post-buckling simulation procedure, and the lattice with the highest strength within each category is used for comparison.


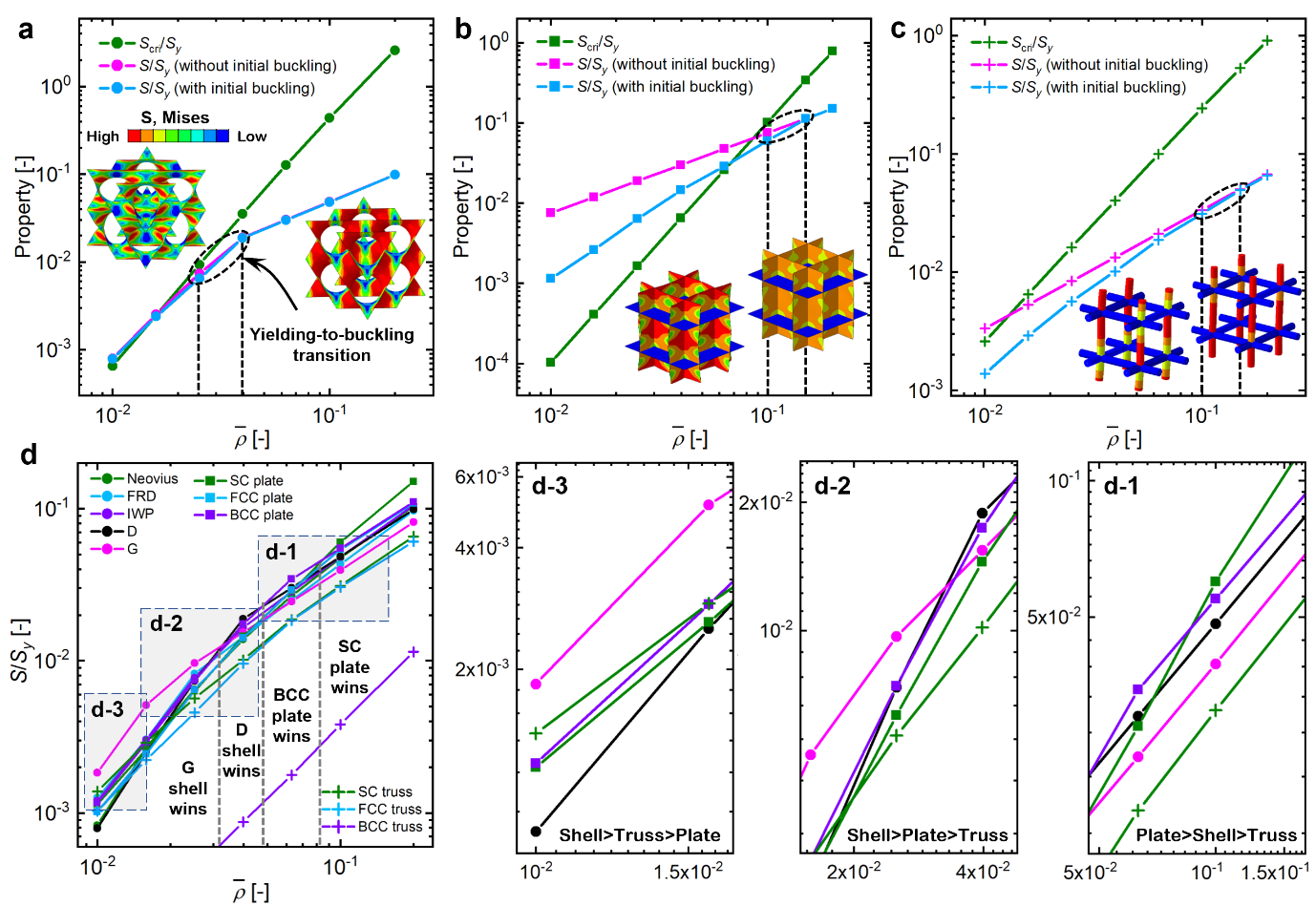


**Figure S1.** Numerical results of effective compressive strength at different RDs: (a-c) Relative critical elastic buckling strength, i.e., the ratio of the critical elastic buckling strength (*S*_cri_) of the lattice to the yield strength (*S_y_*) of the constitutive material, and relative compressive strength (with and without incorporation of initial buckling behaviors) versus RD plots, of (a) D shell lattices, (b) SC plate lattices, and (c) SC truss lattices, respectively. (d) Relative compressive strength versus RD plots of all selected lattices.

**2. Multi-geometry adaptive printing strategy for metallic lattices with low RDs**

All metallic lattices were fabricated by an in-house developed high precision micro laser powder bed fusion (µLPBF) system Hans M100 μ, equipped with an Yb laser source (*λ* = 1.07 μm) and a beam spot size of 25 μm (**Figure S2**). To achieve high accuracy in the three classes of lattices over a wide range of RDs (1.0%-20.0%), substantial improvements have been made in both the hardware and software. The laser beam, layer thickness, and powder size were decreased to improve the printing resolution. A beam expander is utilized to enlarge the size of incident spots, hence facilitating the generation of a reduced beam upon passing through the *f*-theta lens.^[S1]^ To achieve a uniform dispersion of fine powders, a soft rake with an elastic modulus of 40 MPa and a circular cross-section was specifically designed. A low oxygen concentration below 500 ppm was ensured by pumping Nitrogen protective gas. Austenitic stainless steel 316 L (SS316L) with a diameter of 5-25 µm (*D*_50_=16 µm) was selected for printing due to its stable properties and wide applications in various industrial sectors.^[S2]^ The process parameter configurations are listed in Table S1. For the hatch filling, the hatch distance was set as 50 μm and the scanning direction rotated 67° at successive layers.


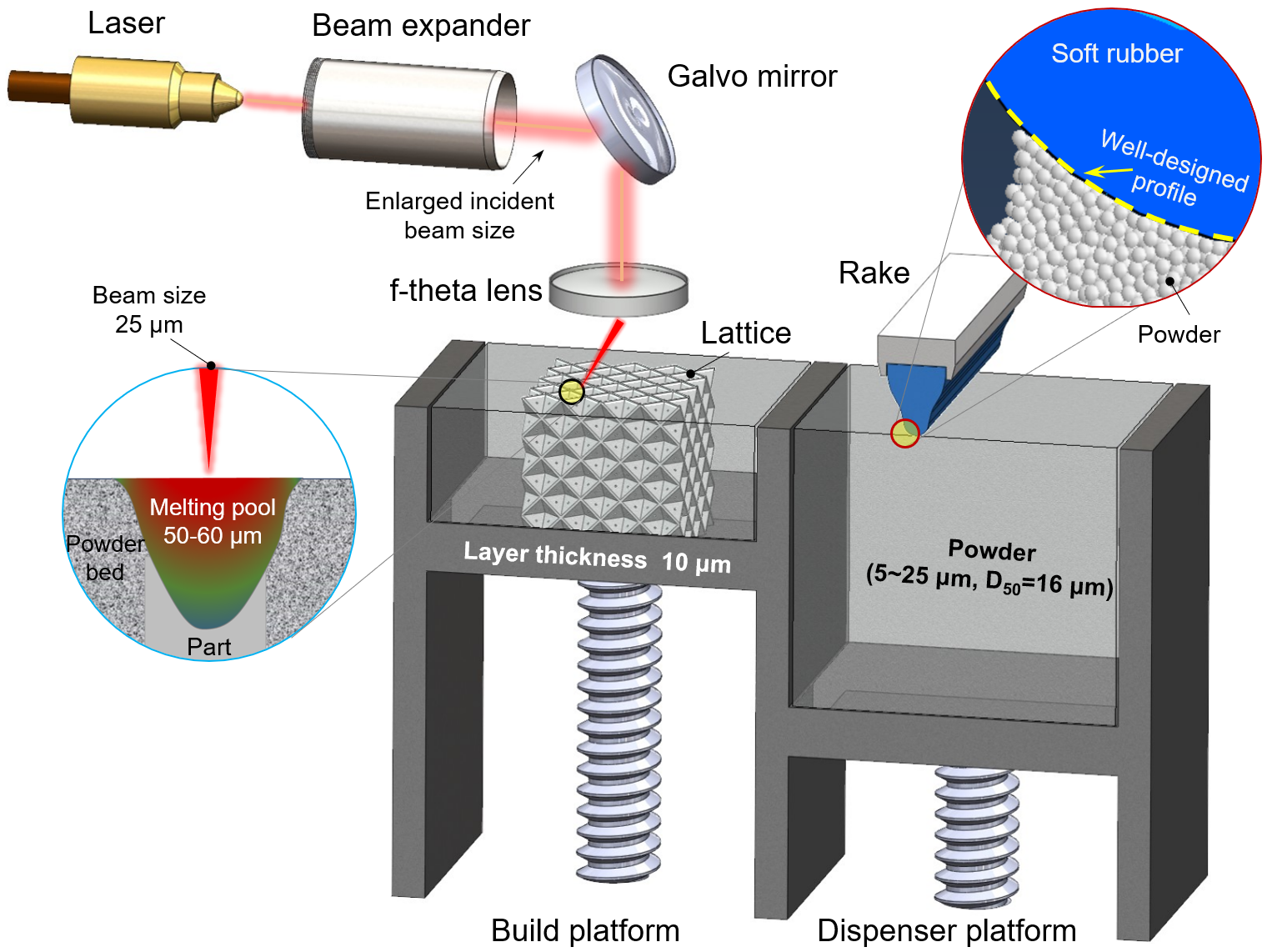


**Figure S2.** Schematic illustration of the µLPBF process.

Despite extensive research on metamaterials, the RDs of most studied metamaterials fall in the middle or high range, typically exceeding 10%. The fabrication of metamaterials with extremely low RDs below 0.1%, on the other hand, is limited to only a few specific types. In contrast, the fabrication of different lattice classes ranging from trusses to shells and plates with low RDs below 10% presents severe challenges, including the lack of precise control of feature sizes, low dimensional accuracy, and inconsistent constitutive material properties. To resolve the problems above, we have developed a multi-geometry adaptive printing strategy for different classes of lattices with low RDs, including optimized building orientations and laser hatching strategies, by which all lattices can be printed precisely with high manufacturing fidelity (**Figure. S3**).

**Table S1.** Process parameter configurations in µLPBF.

| **Parameters** | **Value** |
| --- | --- |
| Laser power | 50 [W] |
| Layer thickness | 10 [μm] |
| Laser spot size | 25 [μm] |
| Hatch angle | 67 [°] |
| Hatch distance | 50 [μm] |
| Scanning speed | 1000 [mms^-1^] |

Specifically, the middle surfaces of TPMS shell lattice models were constructed via an open-source software, Surface Evolver.^[S3]^ Then, various shell lattices with different cell sizes and thicknesses were generated in MATLAB via rescaling and offset of the stereolithography (STL) mesh files. The plate and truss lattices were modeled in the software package SolidWorks 2020. Next, all the geometric models were exported as STL files for slicing. To avoid the large overhang regions of SC plate and truss lattices, the lattices were rotated and printed along [110] and [111] directions, respectively. Afterwards, the samples and supports were cut off from the building plate using electrical discharge machining. Two scanning strategies were employed to fully utilize the high resolution of the printer and decrease the RD of the lattices. As shown in Figure. S3, the single-track scanning along the middle surface was used for ultra-thin walls. In this case, only the middle surfaces of the models were used for slicing to generate the scanning path. Thus, the thickness of the walls was determined by the width of the molten pool, resulting in a thickness of 65.2±4.0 μm for vertical walls. Both hatch filling and border scanning were used for thick walls with thicknesses larger than 100 μm. To ensure the accuracy of circular cross-sections and surface finish, the diameter of the constitutive bars was set as 250 μm. All the hatching path rotates 67° at successive layers.


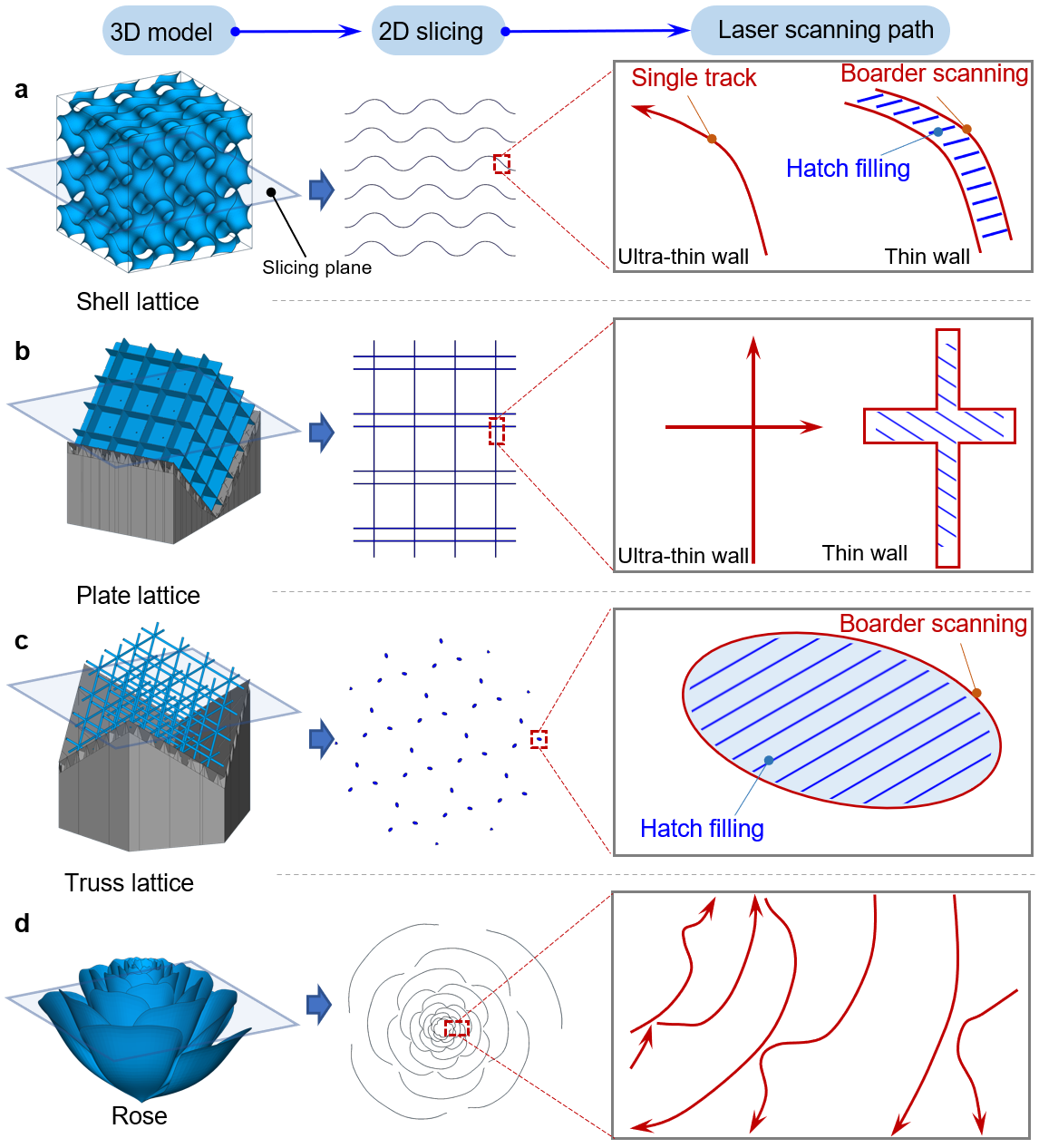


**Figure. S3.** Geometric modelling and scanning path generation by our multi-geometry adaptive printing strategy: (a) shell lattice, (b) plate lattice, (c) truss lattice, and (d) rose.

**3. Experimental characterization**

*3.1 Mechanical properties of constitutive materials*

According to the ASTM E8/E8M standard, the properties of constitutive materials were characterized by uniaxial tensile tests. The stress-strain curves of the constitutive materials, derived from three repeated tests, were shown in **Figure S4**. In this study, G shell lattices with three RDs were employed to illustrate the variation of mechanical response in lattices induced by the variations in stress-strain curves (**Figure S5**). The results, presented in Table S2, indicate that the variations in constitutive materials are sufficiently low, having only a minor impact on the mechanical properties of the lattices under a compressive strain of 1%.

To demonstrate the stability and consistency of the mechanical properties of constitutive materials within the range of interest in this work, thin film dog-bone samples with the thickness of 65.0 μm and bulky dog-bone samples with a thickness of 1.5 mm were used for uniaxial tensile tests (**Figure S6a** and **b**). For this purpose, samples with the dog-bone shape along the thickness direction were designed, of which the gauge range was printed by the single-track path along the longitude direction, while the bulky bars were printed via R67 scanning strategy. Therefore, the thickness of the gauge region was directly dependent on the size of the melting pool, leading to a low thickness of around 65.0 μm. The results show no significant effect of laser scanning strategies on the mechanical properties in terms of yield strength and Vicker hardness (**Figure S6c**). The µLPBF fabricated SS316L samples exhibit a grain size of approximately 7.0-8.0 µm, as well as a sub-grain size of around 309.0 nm, as reported in our prior works. The findings ^[S4]^ indicate a strong correlation between the strength of SS316L and the structural integrity of its sub-grain cell walls, which is attributed to the presence of precipitates, element segregation, and dislocations. In this work, the feature size of samples is significantly larger than the size of sub-grains, and the ratios between the feature size (a thin wall thickness of nearly 65.0 µm) and grain/sub-grain size are approximately 10 and 30, respectively. Regarding plastic properties, the thin film has a smaller elongation compared with the bulk one. The possible reason is that only a limited number of grains exist within thin walls and the large plastic deformation was restricted. In addition, compared with bulk samples, the roughness of the as-printed samples has a more pronounced effect on the fracture behavior. In conclusion, this section demonstrates that the feature size and scanning strategies of R67 for bulk and single track of thin films have no apparent effects on the yield strength of the constitutive materials, and the influence of size effect is negligible within the range of interest in this work.


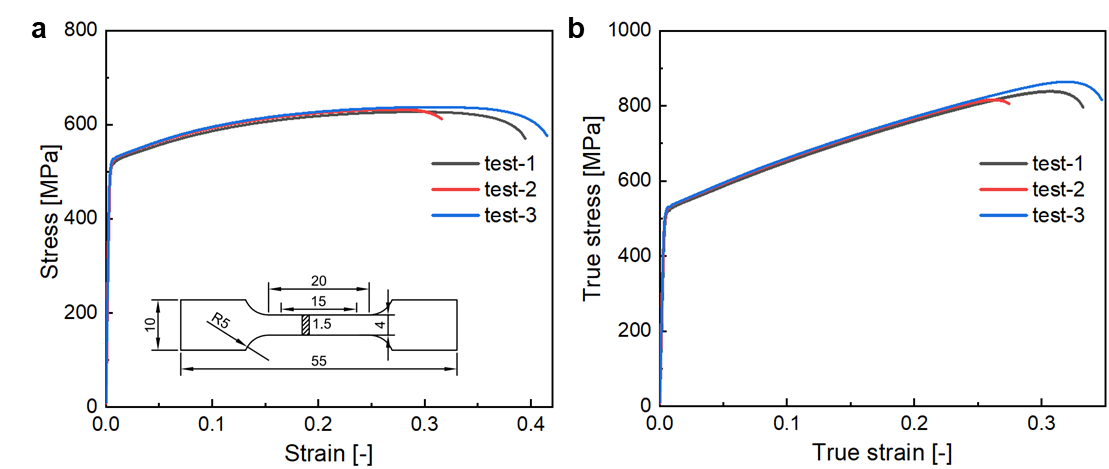


**Figure S4.** Stress-strain curves of SS316L in repeated experimental tests: (a) nominal stress-strain curve, and (b) true stress-strain curve.


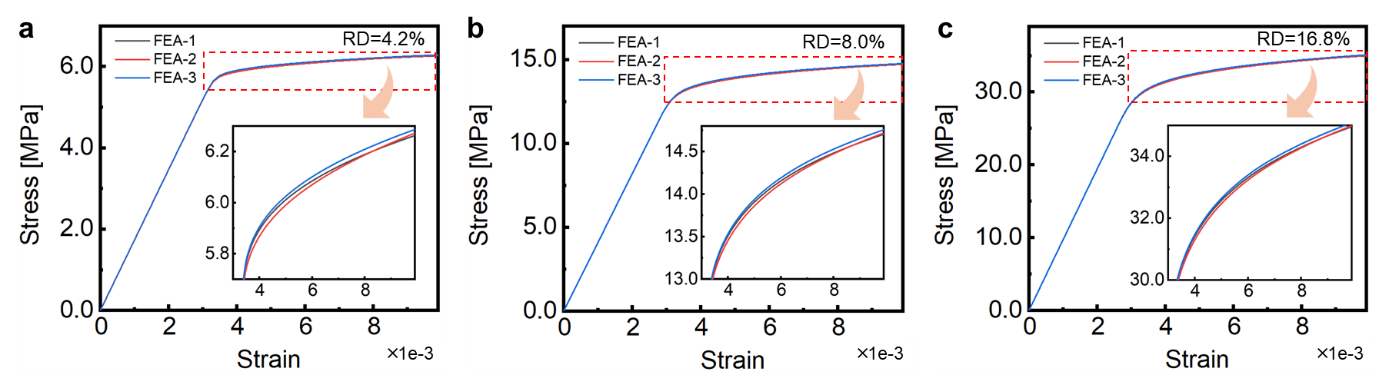


**Figure S5.** Variation of numerical simulation results induced by the deviations of stress-strain curves from tensile tests: (a) G shell at an RD of 4.2%; (b) G shell at an RD of 8.0%, and (a) G shell at an RD of 16.8%.

**Table S2** Relative yield stress of G shell lattices based on the three constitutive stress-strain curves.

| G shell | RD=4.2% | RD=8.0% | RD=16.8% |
| --- | --- | --- | --- |
| FEA-1 | 0.01192 | 0.02685 | 0.06296 |
| FEA-2 | 0.01188 | 0.02677 | 0.06277 |
| FEA-3 | 0.01195 | 0.02692 | 0.06312 |
| Average | 0.0119±3.36e-5(0.28%) | 0.0268±7.51e-5 (0.28%) | 0.063±1.75e-4 (0.28%) |

*Note: The percentage in each bracket shows the absolute value of relative standard deviations.*


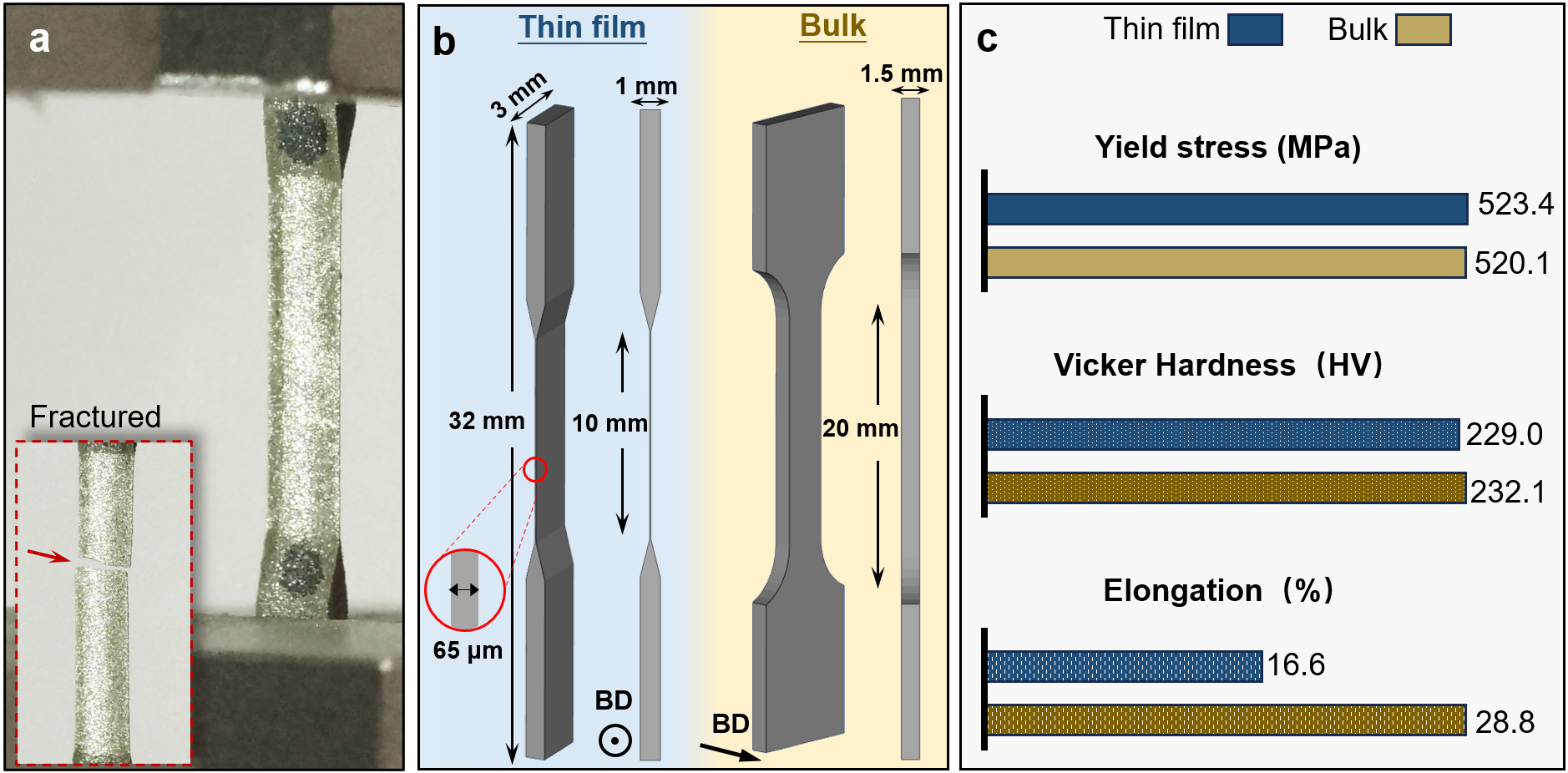


**Figure S6.** Mechanical properties of thin films vs the bulk: (a) dimensions of tensile bars, (b) uniaxial tensile tests of thin films, and (c) comparison of yield stress, Vicker hardness, and elongation between the as-printed thin films and bulk samples.

*3.2 Manufacturing fidelity of different lattices*

For the open-cell lattices: shell and truss, the RD was calculated based on its actual measured weight and bounding box size. To mitigate the potential overestimation of the mass due to adhered particles, the RD of plate lattices was determined based on the unit cell, from which the adhered powders can be effectively eliminated (**Figure S7**). For SC plates with the unit cell sizes of 8 mm and 15 mm, the diameters of the cutting holes on the face centers were set as 400 μm to minimize the loss of material and strength.


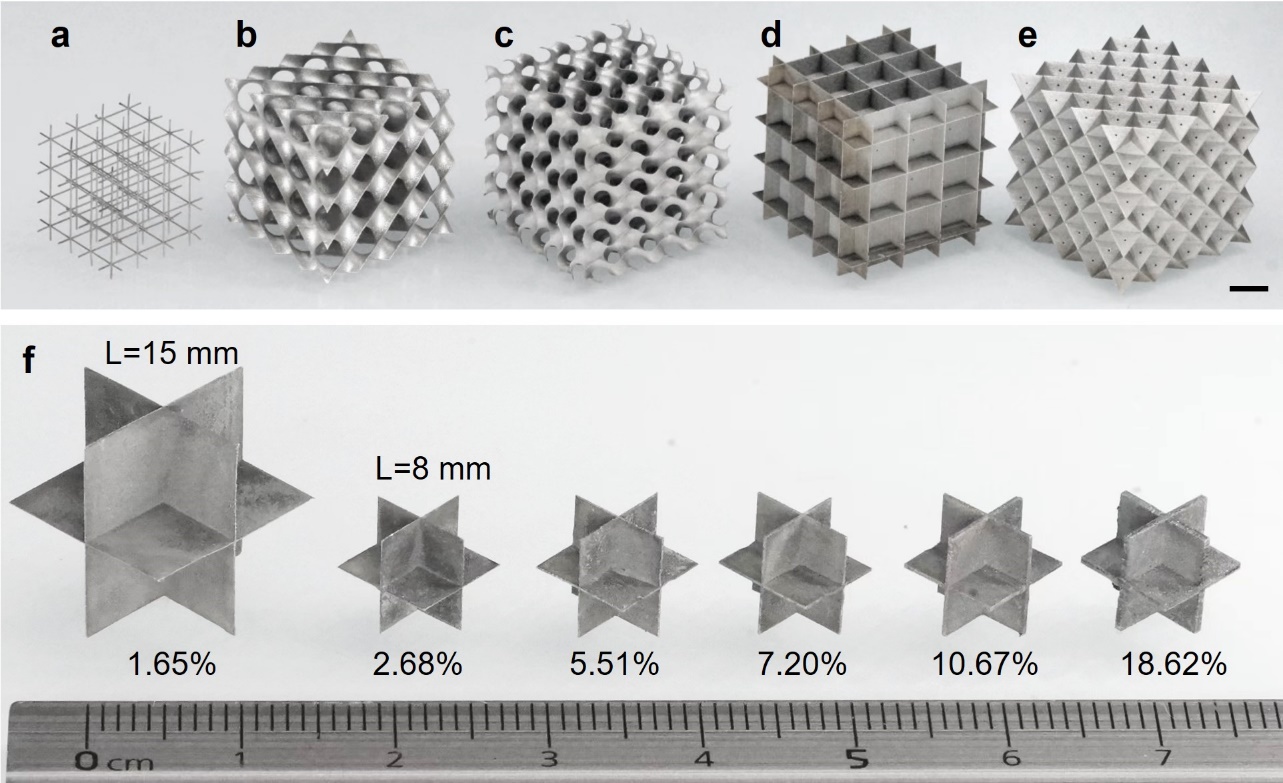


**Figure S7.** As-fabricated lattice samples: (a) SC truss, (b) D TPMS, (c) G TPMS, (d) SC plate, (e) FCC plate, and (f) unit cells of SC plate lattices with different RDs. The scale bar is 10 mm.

The wall thickness and surface quality were investigated using RH-2000 High-Resolution 3D Optical Microscope, HIROX. The average roughness, Ra, was evaluated by the depth-reconstruction via the same microscope. The Micro-CT, Scanco Medical µCT-35 equipped with a 70 kV X-ray source, was used to conduct the non-destructive characterization, then the reconstructed models were exported as STL files to provide the geometric data. As shown in **Figure S8**, the lowest thickness of 65.1 μm with a roughness of 3.1 μm for the vertical thin films can be achieved via the single-track scanning strategy. For the TPMS shell lattice, the shell thickness varies depending on the inclination angle. Here, the unit cells with minimal feature sizes were scanned by micro-CT to validate the high precision of µLPBF. Due to the staircase effect and sintered powders, the thickness increased to about 90.0±12.3 μm with larger variations at lower inclination angles. As the SC plate was rotated by 45° for printing, two types of walls with different printing inclinations appeared, and the mean thicknesses were 65.2 μm and 70.5 μm for vertical and slanted plates, respectively. More powders were sintered in the intersections between walls, but plate lattices still show lower thickness variation than D shell lattices. The roundness and diameter were used to evaluate the printing accuracy of truss lattices. As shown, powders were sintered onto the lower side of the constitutive bars, leading to a reduced roundness and variation of diameter. Therefore, the three classes of lattices were shown to be printed with high manufacturing fidelity using the proposed scanning strategies.


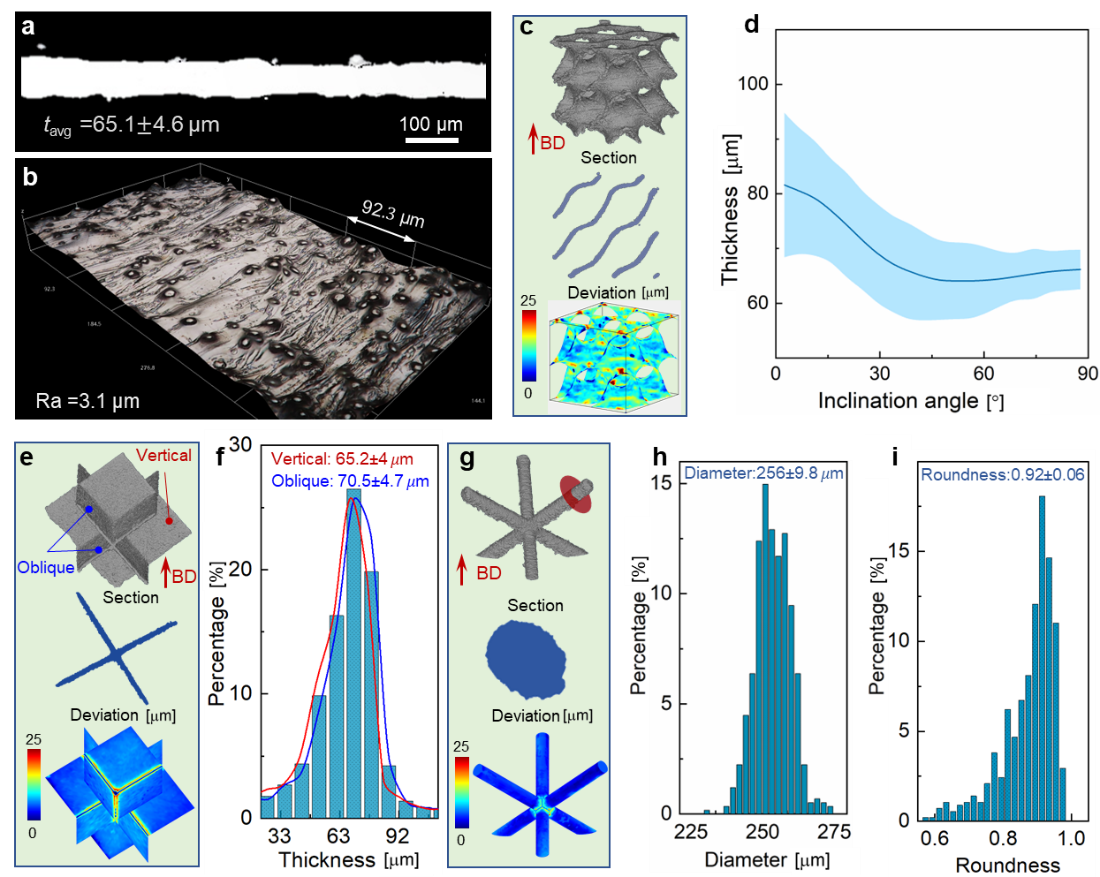


**Figure S8.** Characterizations of manufacturing fidelity: (a) cross-sectional view of vertical thin walls, (b) side surface roughness, (c) micro-CT reconstructed D shell model and its cross-sectional and three-dimensional deviations, (d) thicknesses of D shell lattices at different inclination angles, (e-f) micro-CT reconstructed SC plate model and its thickness distributions, and (g-i) micro-CT reconstructed SC truss model, and the distributions of its cross-sectional diameters and constituent bar roundness. Scale bars are 100 µm in (a), and 92.3 µm in (b), respectively.

The lattice materials fabricated by LPBF exhibit four primary manufacturing defects, thickness variations, holes, surface waviness, and roughness.^[S5]^ The defects that have the most significant impact on the reduction of both linear and nonlinear mechanical properties are surface roughness and thickness variations. These geometric defects can be accurately simulated by reducing the thickness and incorporating the roughness value on each side, thereby improving the accuracy of the simulation.^[S5]^ The micro-CT reconstructed models (Figure S8) indicate that there are no discernible internal defects, such as pores or cracks, present in the lattices. Consequently, our primary emphasis is on identifying and addressing geometric manufacturing flaws. The current version of numerical simulations incorporates manufacturing faults by accounting for thickness/diameter reduction, which allows for the assessment of the mechanical response variation.

Initially, we conducted measurements of the surface roughness at different inclination angles, including: 0°, 25°, 30°, 35°, 40°, 45°, 50°, 55°, 60°, 65°, 70°, 75°, 80°, 85°, 90°. These measurements are depicted in **Figure S9**. The top side exhibits a low surface roughness (Ra) of less than 5 μm, whereas the bottom side has a high surface roughness with greater variations. Therefore, the reduction value was determined by taking the average Ra value of both the upside and downside surfaces, and subsequently incorporating it into the numerical simulations. In this case, we utilize the G shell as an illustration to demonstrate the outcomes of including manufacturing defects at three different RDs. The G shell encompasses a wide range of inclination angles, which accurately represents the intricate distribution of these defects.

Three different relative densities of G shell lattices were chosen: 4.2%, 8.0%, and 16.8%. These shells have the same unit cell size of 4 mm and average wall thicknesses of 64.3 μm, 110.8 μm, and 220.7 μm, respectively (**Figure S10**). The results suggest that adding defects through thickness reductions based on surface roughness is an effective method to improve numerical accuracy. When analyzing the numerical data, the presence of defects led to a decrease in compressive strength by 27%, 14%, and 8%, respectively. As the wall thickness increases, the impact of defects on the strength decreases.


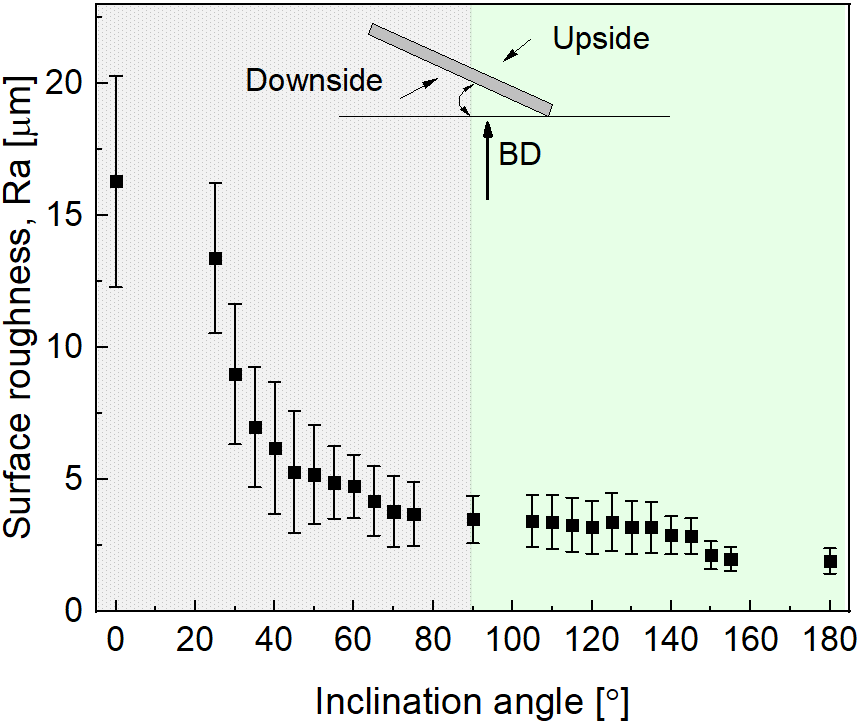


**Figure S9.** Surface roughness at different orientations.


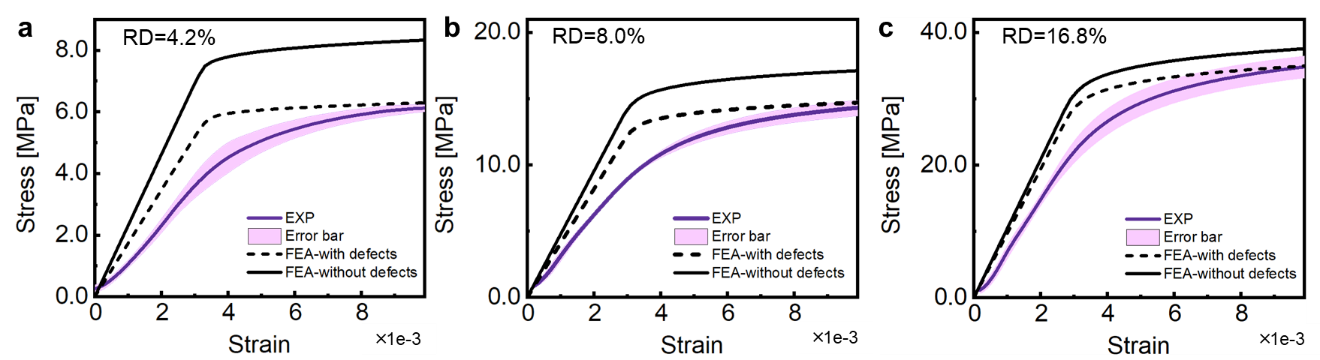


**Figure S10.** Comparison of experimental and numerical results with and without implementation of defects: (a) G shell at an RD of 4.2%; (b) G shell at an RD of 8.0%; (c) G shell at an RD of 16.8%.

*3.3 Compression tests*

Quasi-static compression tests were conducted on an MTS universal test machine (Model 370.1, MTS Landmark® Testing Solutions) to measure the mechanical properties of the lattice samples. To measure the samples’ Young’s moduli accurately, the range of the applied force was set below the yield strength, ensuring the deformation was within linear elastic regime during loading and unloading tests. The loading and unloading rate were set as 50 N/s, and the modulus was calculated according to the unloading curves. Usually, the first loading curve shows a lower slope, which is mainly attributed to the non-flat surfaces and sintered powders. As the unloading curve has better stability than the loading curve, the force-displacement slope of the samples, *k_ms_*, was calculated from 3 repeated unloading curves. Moreover, the stiffness of the machine, which was obtained from compression tests of the machine fixtures (without samples), was also considered to eliminate the effects of machine deformations on the moduli of the samples. The force-displacement slope of the machine, *k_m_*, was calculated from the unloading curves of the machine fixtures. Finally, the sample’s Young’s modulus was calculated as:

| $E=\frac{hk_{ms}k_{m}}{A(k_{m}-k_{ms})}$ | (S1) |
| --- | --- |

where *A* and *h* denote the cross-section area and height of the sample, respectively. Moreover, the compressive strain rate was set as 1.0×10^-3^ s^-1^ for large strain compression tests, and the compressive strength of various samples was calculated as the 0.2% offset strength from the stress-strain curves.

The numerically evaluated and experimentally measured relative Young’s modulus (*E*/*E_s_*) versus RD ($\bar{\rho}$) plots of the lattices are shown in **Figure S11**. The numerical results show that incorporating initial buckling behavior has no effect on the stiffness of the lattices, and the relative Young’s moduli of all investigated lattices possess no sudden drops (Figure S11a). At the same RD, SC plate lattices and BCC truss lattices possess the highest and lowest Young’s moduli, respectively, FCC truss lattices possess the second lowest moduli, while the other lattices are within the middle range. However, the experimental results show that the measured Young’s moduli decrease significantly with decreasing RDs (**Figure S11b**), showing an inconsistent trend with numerical simulations. The difference between the numerical and experimental results may be attributed to the local buckling induced during the flattening of lattice samples with lower RDs in experimental tests, which makes the measured Young’s moduli lower than the actual values. Comparatively, SC plate lattices and G shell lattices possess the highest Young’s moduli at higher and lower RD regimes, respectively.


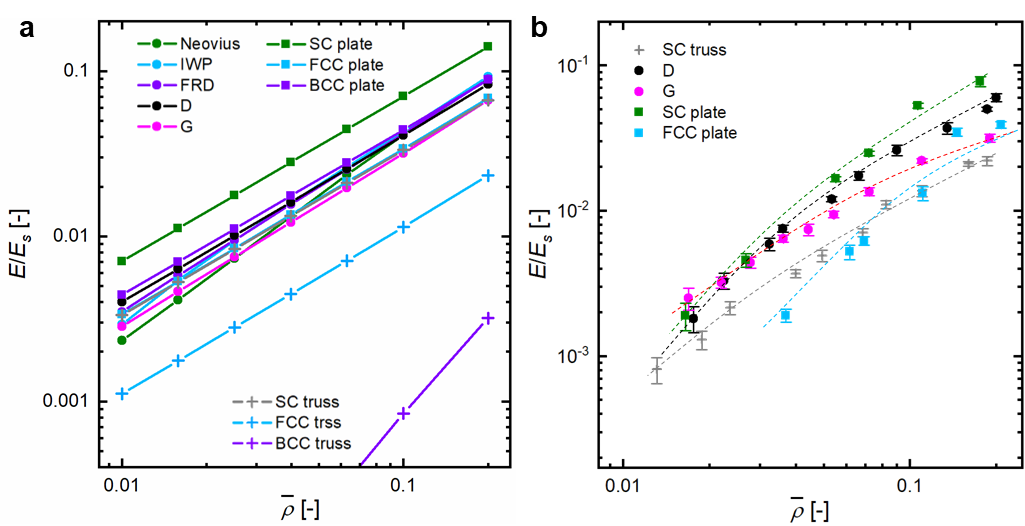


**Figure S11.** Relative Young’s modulus (*E*/*E_s_*) versus RD ($\bar{\rho}$) plots: (a) numerical results of the 11 selected types of lattices, and (b) experimental results of D, G shell, SC, FCC plate, and SC truss lattices.

3.4 Repeatability of mechanical response

**Figure S12** displays the compressive stress-strain curves and snapshots from three different relative densities of lattice materials, providing evidence of good repeatability in their mechanical response. The relative density of all lattices varies by less than 1.5 percent. Specifically, all the lattices at different RDs exhibit a low level of variation in their stress-strain curves, with only a modest rise in variation at large strain ranges, where the lattice typically demonstrates a high level of non-linear deformation behavior. The detailed data, including relative density (RD), relative yield stress (S/Sy), and relative plateau stress (PS/Sy), are listed in Table S3. In general, the variation of relative compressive strength is less than 5% for the three different types of lattices over the three RD ranges, demonstrating a high level of experimental repeatability.


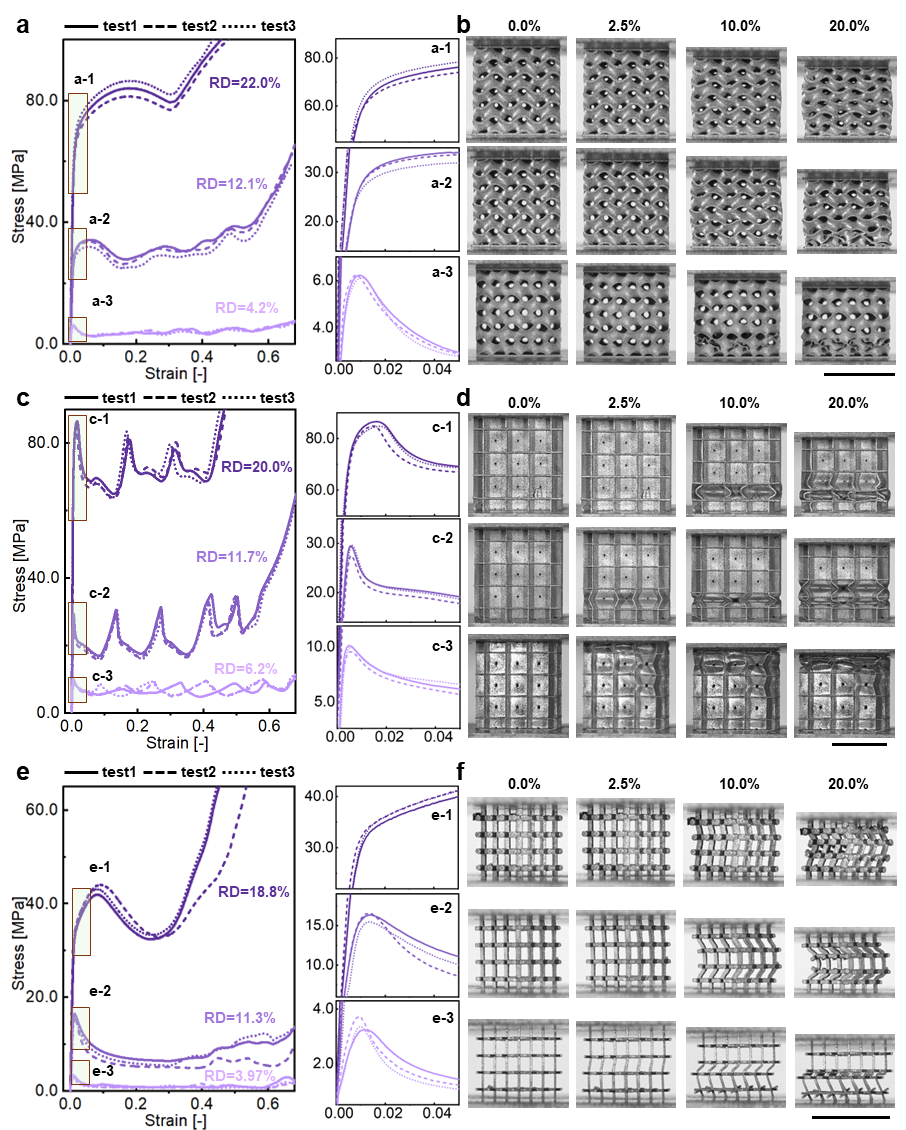


**Figure S12.** Compressive stress-strain curves and snapshots of the three types of lattices at low, moderate, and high RDs: (a-b) G shell, (c-d) SC plate, and (e-f) SC truss lattices. The scale bars are 10mm in (b, d, f).

Table S3 Mechanical properties of the three types of lattices with different RDs.

| Lattice materials | RD  [%] | *S*/*S_y_*  [-] | *PS*/*S_y_*  [-] |
| --- | --- | --- | --- |
| G shell | 4.22±0.04 (0.9%) | 0.0113±3.0e-4 (2.6%) | 0.008±1.8e-4 (2.3%) |
|  | 12.10±0.08 (0.7%) | 0.0521±1.8e-3 (3.6%) | 0.06±2.7e-3 (4.5%) |
|  | 22.02±0.13 (0.6%) | 0.1171±3.6e-3 (3.1%) | 0.17±5.7e-3 (3.4%) |
| SC plate | 6.23±0.08 (1.3%) | 0.0189±5.0e-4 (2.6%) | 0.0136±3.2e-4 (2.4%) |
|  | 11.70±0.163 (1.4%) | 0.0538±2.0e-3 (3.7%) | 0.0437±1.1e-3 (2.5%) |
|  | 19.97±0.24 (1.2%) | 0.1540±2.2e-3 (1.42%) | 0.1418±7.2e-3 (5.1%) |
| SC truss | 3.97±0.017 (0.4%) | 0.0062±1.9e-4 (3.1%) | 0.0025±3.2e-4 (12.8%) |
|  | 11.30±0.08 (0.7%) | 0.03±6.2e-4 (2.1%) | 0.0159±2.2e-3 (13.8%) |
|  | 18.82±0.11 (0.6%) | 0.062±1.0e-3 (1.6%) | 0.09±1.5e-2 (16.7%) |

*Note: The percentage in each bracket shows the absolute value of relative standard deviations.*

**4. Elastic anisotropy of different types of lattices**

The mechanical anisotropy of the lattices varies depending on the lattice type and RD. Herein, the elastic anisotropy of G, D shell, SC plate and truss lattices was investigated (**Figure S13**). In general, G shell lattices show the lowest degree of anisotropic elasticity, whose maximum to minimum moduli ratios are within the range of [1.03, 1.12] at 1.0-20.0% RDs. Comparatively, SC plate lattices possess a higher degree of anisotropic elasticity, with a higher moduli ratio of 2.0. In contrast, the Young’s modulus along the stiffest direction ([100]) of SC truss lattices outperforms that along the softest direction ([111]) by approximately 12-166 times, and the Young’s modulus decreases substantially when the loading direction deviates from [100] direction. Overall, these results further demonstrate the superior lightweight potentials of G shell lattices, which possess high compressive strength with a low degree of anisotropic elasticity at low RD regimes.


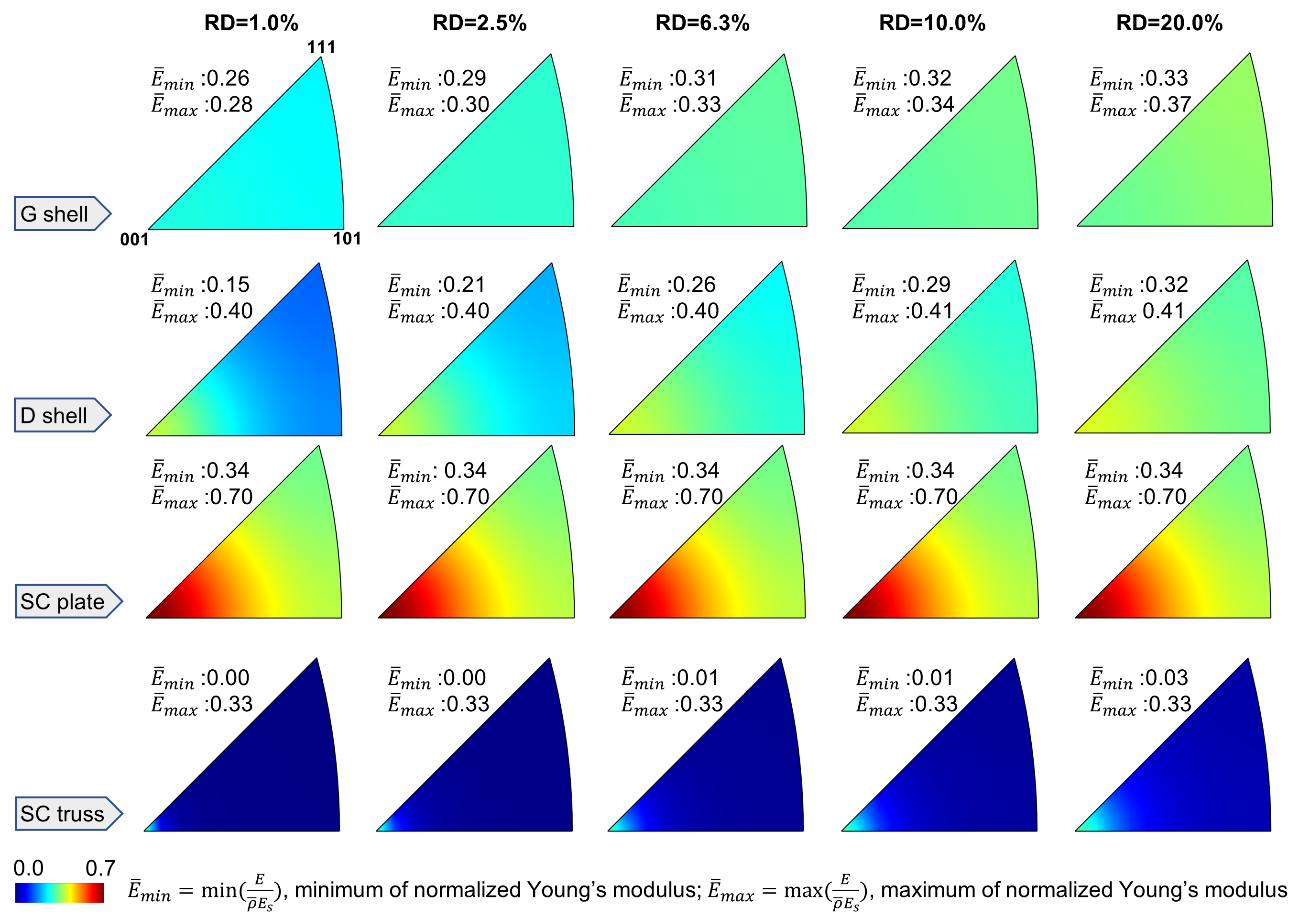


**Figure S13.** Anisotropy of the normalized Young’s moduli of G shell, D shell, SC plate, and SC truss lattices with different RDs.

**5. Bending and membrane strain energy partition for lattices**

First, we divided the stress (σ) of plate/shell lattices into the membrane term, σ^(^*^m^*^)^, and bending term, σ^(^*^b^*^)^, according to the Kirchhoff-Love plate/shell theory. To precisely perform the partition, an elastic material was adopted for analysis. On the middle surface, the bending stress, σ^(^*^b^*^)^=0 and the total stress is exactly equal to the membrane stress σ^(^*^m^*^)^. Otherwise, the bending stress σ^(^*^b^*^)^ ≠0, and the total stress is equal to the sum of the membrane stress, σ^(^*^m^*^)^, and bending stress σ^(^*^b^*^)^. On the top/bottom surface, the bending stress, σ^(^*^b^*^)^, reaches the peak value. Therefore, the total stress of the top/bottom surface deviates the most from the membrane stress. The membrane stress terms of the lattices are:

| $\sigma_{11}^{\left( m \right)}=\frac{{SF}_{1}}{\delta}$  $\sigma_{22}^{\left( m \right)}=\frac{{SF}_{2}}{\delta}$  $\sigma_{12}^{(m)}=\frac{{SF}_{3}}{\delta}$ | (S2) |
| --- | --- |

where δ is the shell thickness, *SF_1_*, *SF_2_*, *SF_3_* are the normal and shear stress resultants within the tangent plane of the shell mid-surface, respectively. The bending stress terms of the lattices are:

| $\sigma_{11}^{\left( b \right)}=12\frac{{SM}_{1}}{\delta^{3}}z$  $\sigma_{22}^{\left( b \right)}=12\frac{{SM}_{2}}{\delta^{3}}z$  $\sigma_{12}^{(b)}=12\frac{{SM}_{3}}{\delta^{3}}z$ | (S3) |
| --- | --- |

where *z* denotes the coordinate along the normal direction and satisfies -δ/2≤*z*≤δ/2. Therefore, the membrane strain energy and bending strain energy of plate/shell lattices can be calculated by the following equations:

| ${SE}^{(m)}=\frac{\delta}{2E}\iint_{A} \left\{ \left[ \sigma_{11}^{\left( m \right)} \right]^{2}+\left[ \sigma_{22}^{\left( m \right)} \right]^{2}-2\nu\sigma_{11}^{\left( m \right)}\sigma_{22}^{\left( m \right)}+2(1+\nu)\left[ \sigma_{12}^{\left( m \right)} \right]^{2} \right\}dA$  ${SE}^{(b)}=\frac{6}{E\delta^{3}}\iint_{A} \left[ \left( {SM}_{1} \right)^{2}+\left( {SM}_{2} \right)^{2}-2\nu\left( {SM}_{1}\cdot{SM}_{2} \right)+2\left( 1+\nu\right)\left( {SM}_{3} \right)^{2} \right]dA$ | (S4) |
| --- | --- |

Similarly, the strain energy of truss lattices can be partitioned into the membrane term and bending term via the following equations:

| $SE^{(m)}=\int_{L} \frac{(\sigma_{11}^{(m)})^{2}A}{2E}dL$  $SE^{(b)}=\int_{L} \frac{(\sigma_{11}^{(b)})^{2}I}{2ER^{2}}dL$ | (S5) |
| --- | --- |

where *R*, *A*, and *I* denote the radius, area, and moment of inertia of the cross section, $\sigma_{11}^{(m)}$ denote the membrane normal stress, and $\sigma_{11}^{(b)}$ denote the maximal bending normal stress on the cross section. Afterwards, the bending strain energy ratio (BSER) of the lattices is defined as:

| $BSER={SE}^{\left( b \right)}/({SE}^{\left( m \right)}+{SE}^{(b)})$ | (S6) |
| --- | --- |

The BSERs of FCC plate and D shell lattices with different RDs are illustrated in **Figure S14**.


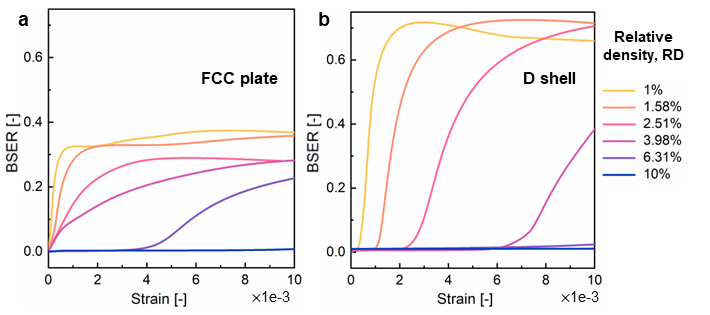


**Figure S14.** The BSERs of (a) FCC plate, and (b) D shell lattices with different RDs.

Variations in buckling strength and deformation patterns among different types of lattices are influenced by their distinct buckling modes, and SC plate shows a potential transition between the first-order and second-order buckling modes under specific conditions.

Herein, using lattices with an RD of 1% as an example, the displacement fields of the first three orders of buckling modes are depicted in **Figure S15**. For the SC plate lattice, the first-order and second-order buckling modes primarily differ in wavelength, with the central regions bulging out in opposite directions, as indicated in Figure S15a. In comparison, the shell lattice structures exhibit three modes with distinct deformation patterns and varying levels of stress concentration, with these deformation patterns occurring mutually exclusively. While the SC truss lattice displays similarities in the first-order and second-order buckling modes, the maximum displacements occur in opposite directions in different planes. Due to the simplicity of the SC configuration, once the initial buckling mode occurs, subsequent deformations tend to follow this pattern and are less likely to transition to alternative patterns.

However, for the SC plate lattice, if the face in the central region (R-II) buckles in the opposite direction, the first-order mode can transit to the second-order mode. **Figure S16a** illustrates the displacement field of the SC plate at various compressive strains, and a possible evolution process of buckling modes is schematically demonstrated in Figure S16b. Upon initial compressive loading, the vertical wall buckles to the left following the first-order buckling mode. As the load increases, the second-order mode is likely to manifest, causing the face to move in the reverse direction. Consequently, the edges near the joint regions move in the opposite direction, while the central regions maintain their original displacement directions due to the larger degree of deformation that is hard to reverse. This results in the formation of a hybrid deformation pattern, where the central regions retain the first-order mode configuration, while the joint regions transit to the second-order mode configuration. This evolution was also captured during compressive test experiments (Movie S1, Supporting Information).


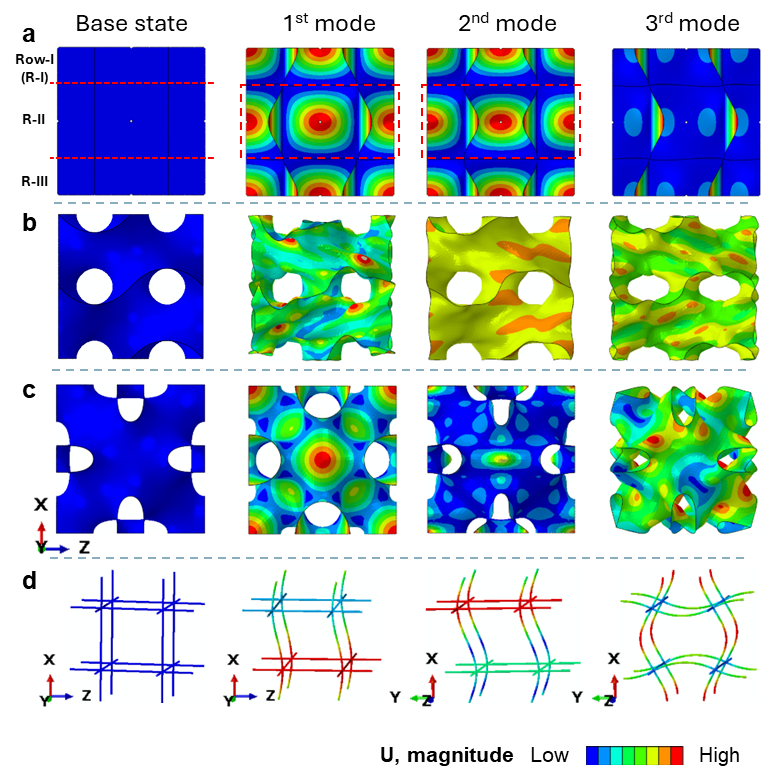


**Figure S15.** Different buckling modes of various lattice structures under compressive loading along the *x*-axis: (a) SC plate; (b) G shell; (c) Neovius shell. (d) SC truss.


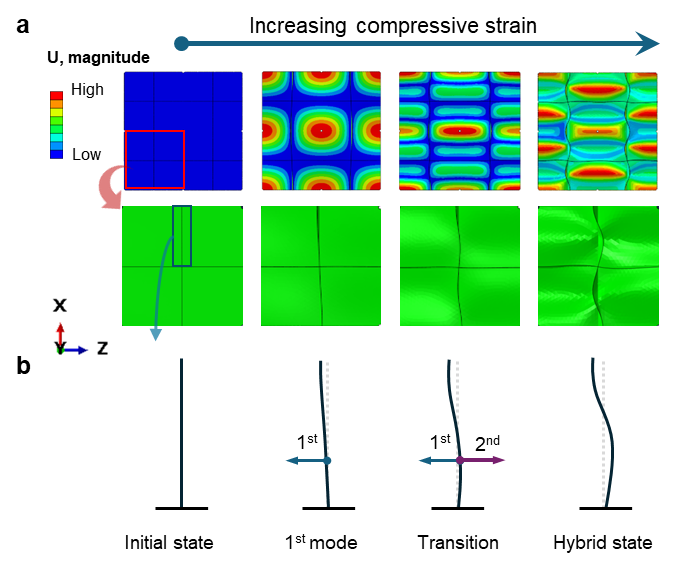


**Figure S16.** Buckling mode evolution of SC plate lattice: (a) Displacement field of the SC plate at various compressive strains along the *x*-axis; (b) possible evolution process of buckling modes.

**6. Cumulative distribution of strain energy density**

The normalized strain energy density (NSED), which is defined as the local strain energy density $\psi$ divided by the macroscopic average strain energy density $\bar{\psi}$, was used to characterize the strain energy distribution of the lattices. The cumulative distribution function, which refers to the probability that the NSED is less than or equal to a specified value, is employed to quantitatively assess the mechanical efficiency and structural stability of the lattices under compressive loading. The cumulative distributions of the NSEDs of SC plate, G shell, and SC truss lattices with 2.51% RD are shown in **Figure S17a-c**. At low strains, the NSEDs of SC plate and truss exhibit a characteristic step function distribution. Since only 2/3 and 1/3 elements bear external loads and store the strain energies of SC plate and truss, the curves exhibit a distinct step at the NSED values of 1.5 and 3, respectively (Figure S17a and c). Nevertheless, this configuration represents an unstable state for both lattices, due to their susceptibility to structure buckling failure. The step function exhibits a transition towards a more gradual profile, implying the reorganization of strain energy and an escalation of stress concentration. For instance, considering the NSED value at a cumulative distribution function of 99%, a notable increase is observed in SC plate and truss lattices when the strain is augmented from 0.01% to 1%: the NSED of the SC plate increases by 296.9%, rising from 1.6 to 6.35, while that of the SC truss increases by 52.8%, advancing from 2.99 to 4.57. Furthermore, the SC plate lattice demonstrates a pronounced sensitivity to compressive strain, manifesting a swift evolution in curve profile starting from a low strain of 0.01%. Conversely, the SC truss exhibits a relatively high level of stability, with the curve remaining almost unchanged below a compressive strain of 0.5%, as illustrated in the magnified inset in Figure S17c. Noteworthy is the fact that the maximum NSED of the SC plate at a compressive strain of 0.01% already reaches 5.227, indicating a notable degree of stress concentration. In contrast, the G shell exhibits superior structural stability and minimal stress concentration. The cumulative distribution curve of the G shell remains almost unaltered within a compressive strain of 0.5%, with the maximum NSED experiencing only a 20% increase from 2.34 to 2.84 as the strain escalates from 0.01% to 1% (Figure S17b). The extra flexural deformation in SC plate and truss occurs rapidly upon buckling, which leads to a significant increase in bending effects. To capture this rapid transition induced by buckling, the strain energy is further divided into membrane and bending terms for more detailed investigation.


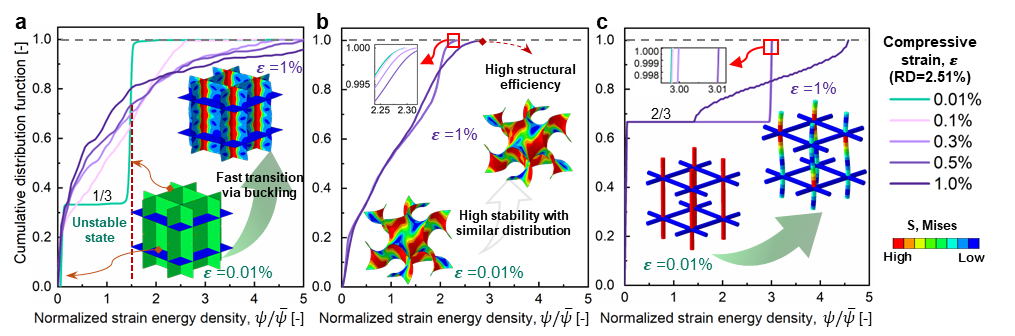


**Figure S17.** Cumulative distribution of strain energy within: (a) SC plate, (b) G shell, and (c) SC truss lattices with 2.51% RD.

**7. Construction of corrugated geometry and geometric noise**

*7.1 Corrugated SC plate*

The corrugated SC plate was constructed via offsetting the middle surface along the normal direction. The trigonometric function was employed to provide the periodically distributed offset value. For instance, the offset value, ∆*d_z_*, along $z$ direction for the XY planes is determined by the following function:

| ${\Delta d}_{z}=H\sin\left( \omega x \right)\sin\left( wy \right)$ | (S7) |
| --- | --- |

where $H$ is the maximum offset value, $\omega={2\pi}/{L_{c}}$, *L_c_* is the periodic length. In this case, *L_c_* =*L* and *H*=0.03*L*, where *L* is the unit cell length of the SC plate (**Figure S18a**). The surface area only increased by 0.9% after introducing the corrugation.

*7.2 Geometric noise*

The random geometric noise is used to mimic the distribution of actual random geometric defects (**Figure S18b**), and the offset value, ∆*d*, is normally distributed (**Figure S18c**) and calculated by the following equation:

| $\Delta d=H\frac{X}{X_{max}}$ | (S8) |
| --- | --- |
| $X\sim\mathcal{N}\left( \mu,\sigma\right)$ | (S9) |

where *H* is the maximum offset value and is equal to 0.1% of the unit cell length, the random variable *X* is normally distributed with the mean value μ=0 and standard deviation σ=1, and *X_max_* is the maximum value for a given set of *X*. The following function evaluates the dimensionless geometric noise, $\bar{GN}$, of the unit cell:

| $\bar{GN}=\frac{\sum_{i=1}^{n} {\Delta d}_{i}^{2}}{L^{2}}$ | (S10) |
| --- | --- |

where ∆*d_i_* denotes the offset value at the *i*-th mesh vertex and *i*∈[1, *n*].


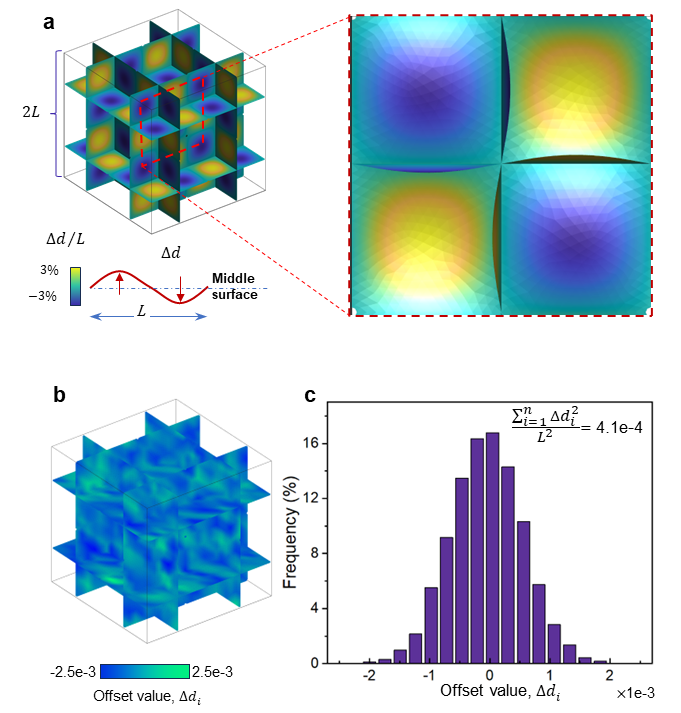


**Figure S18.** Illustration of the imperfections and geometric noises of SC plate lattices. (a) Regular corrugated SC plate lattice, (b) random geometric noise, and (c) the frequency distribution of noises.

*7.3 Corrugated SC truss and shell*

The corrugation was introduced into SC truss lattices via offsetting the axis of rod in the form of a sine function with the same wavelength as that in Equation S7. For shell lattices, due to their curved nature, the corrugation was introduced in the regions with low net curvatures, as shown in **Figure S19**.

Finally, we use the strain energy at 0.1% strain to evaluate the stability of the initial state of original SC plate, corrugated SC plate, and G shell lattices, in which *ϕ*, *ϕ_0_* denote the strain energies of the lattices with and without geometric noises, respectively (**Figure S20**). The results clearly demonstrate that original SC plate lattices are highly unstable, while their stability can be significantly enhanced via the introduction of geometric imperfections. In contrast, G shell lattices exhibit inherently superior stability under compression.


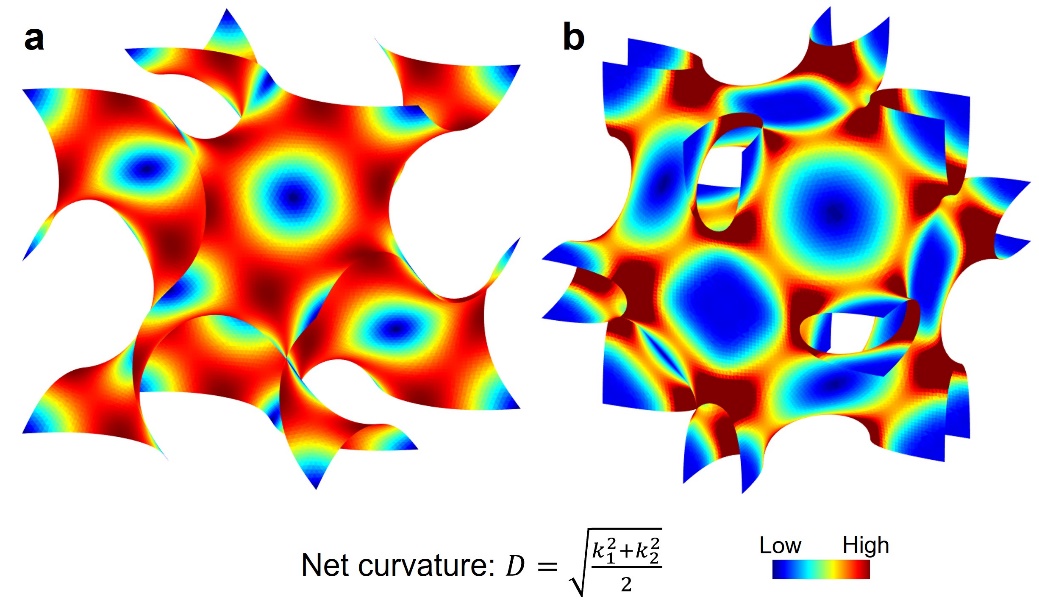


**Figure S19.** Surface net curvatures of (a) G shell and (b) Neovius shell mid-surfaces. The net curvature, *D*, is calculated based on the minimum (*k*_1_) and maximum (*k*_2_) principal curvatures.


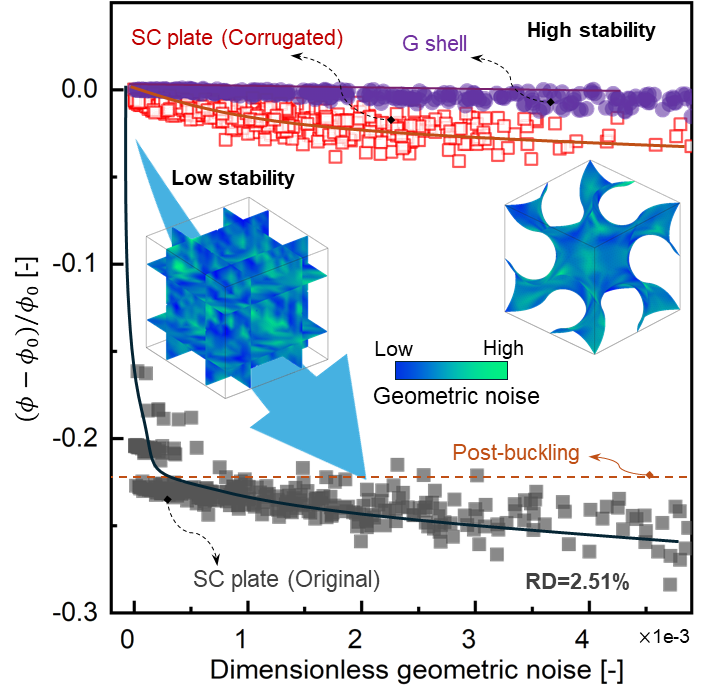


**Figure S20.** Sensitivity of the strain energies of original SC plate, corrugated SC plate, and G shell lattices on the geometric noise. *ϕ* and *ϕ_0_* denote the strain energies of the lattices with and without geometric noises, respectively.

**8. Effects of aspect ratio on buckling strength**

The aspect ratio is a critical factor that determines the buckling strength of a structural member. At meso-scale, we investigate the effects of aspect ratio on the critical elastic buckling strength (*S_cri_*) of the lattices. Herein, the aspect ratio of plate/shell lattices is defined as the ratio of unit cell size to wall thickness, while the ratio of unit cell size to cross-sectional diameter is used to describe the aspect ratio of truss lattices (**Figure S21a**). The relative area of the plate/shell mid-surface is denoted as *A*/*D*^2^, where *A* and *D* denote the plate/shell mid-surface area and unit cell size, respectively. The RD ($\bar{\rho}$) and aspect ratio (*r*) of plate/shell lattices are related as:

| $\overline{\rho}=\frac{At}{D^{3}}=\frac{A}{D^{2}}\frac{1}{r}$ | (S11) |
| --- | --- |

where *t* denotes the wall thickness, and *r* is the aspect ratio, *D/t*. The value of *A*/*D*^2^ is constant for a specified plate/shell lattice, and the specific values of D, G, IWP, Neovius, FRD shell lattices, and SC, FCC, BCC plate lattices are 3.84, 3.09, 3.47, 3.51, 4.77, 3.0, 6.93 and 8.49, respectively. According to Equation (S11), the RD of plate/shell lattices varies in accordance with the aspect ratio by an inverse proportional relationship. Furthermore, the RD and aspect ratio of truss lattices are related as:

| $\overline{\rho}=\frac{\pi Ld^{2}}{4D^{3}}=\frac{\pi L}{4D}\frac{1}{r^{2}}$ | (S12) |
| --- | --- |

where *d* is the diameter of the constitutive bar, and *r* is the aspect ratio, *D/d.* The ratio of the total bar length *L* to unit cell size *D* is constant for a specified truss lattice, and the specific values of *L*/*D* for SC, FCC, and BCC truss lattices are 3.0, 16.97, and 6.93, respectively. According to Equation (S12), the RD of truss lattices varies in accordance with the aspect ratio by an inverse square relationship. The plots of RD versus aspect ratio of the three classes of lattices are illustrated in **Figure S21b**. In practice, the RD and aspect ratio are geometric descriptions of the same lattice materials from two different perspectives, in which the former represents the homogenized volume fraction of the lattice unit cell, whereas the latter describes the specific feature size of internal microarchitectures.

The relative critical buckling strength (*S*_cri_/*S_y_*) versus aspect ratio plots of shell, plate, and truss lattices are illustrated in **Figure S21c-e**. The buckling strength of all investigated lattices decreases monotonically with rising aspect ratios, in which truss lattices exhibit a much more significant decreasing trend than plate and shell lattices. For a specified lattice, the RD varies in accordance with the aspect ratio by an inverse proportional (plate/shell lattices) or inverse square (truss lattices) relationship, therefore the effects of aspect ratio and RD on the buckling strength show an opposite trend. Various types of lattices with identical relative densities typically manifest distinct aspect ratios. Despite sharing the same relative density, the aspect ratio has been shown to significantly influence the buckling strength (**Figure S21f-h**). In comparison, the buckling strength of plate lattices exhibits minimal variations across different aspect ratios, while that of truss and shell structures demonstrates significant sensitivity to the aspect ratio. In general, the relationship between buckling strength and aspect ratio follows a non-monotonic trend, as lattices with smaller aspect ratios do not consistently display superior buckling strength given the same relative density. This suggests that, in addition to aspect ratio and relative density, other factors such as topological configurations also play a significant role in influencing the buckling strength. Overall, plate lattices exhibit lower buckling strength compared to truss and shell lattices. A trend where the buckling strength of truss lattices gradually surpasses that of shell lattices as the relative density decreases can also be observed. This indicates that lattices with lower aspect ratios are more likely to achieve exceptional buckling strength at ultra-low relative densities.


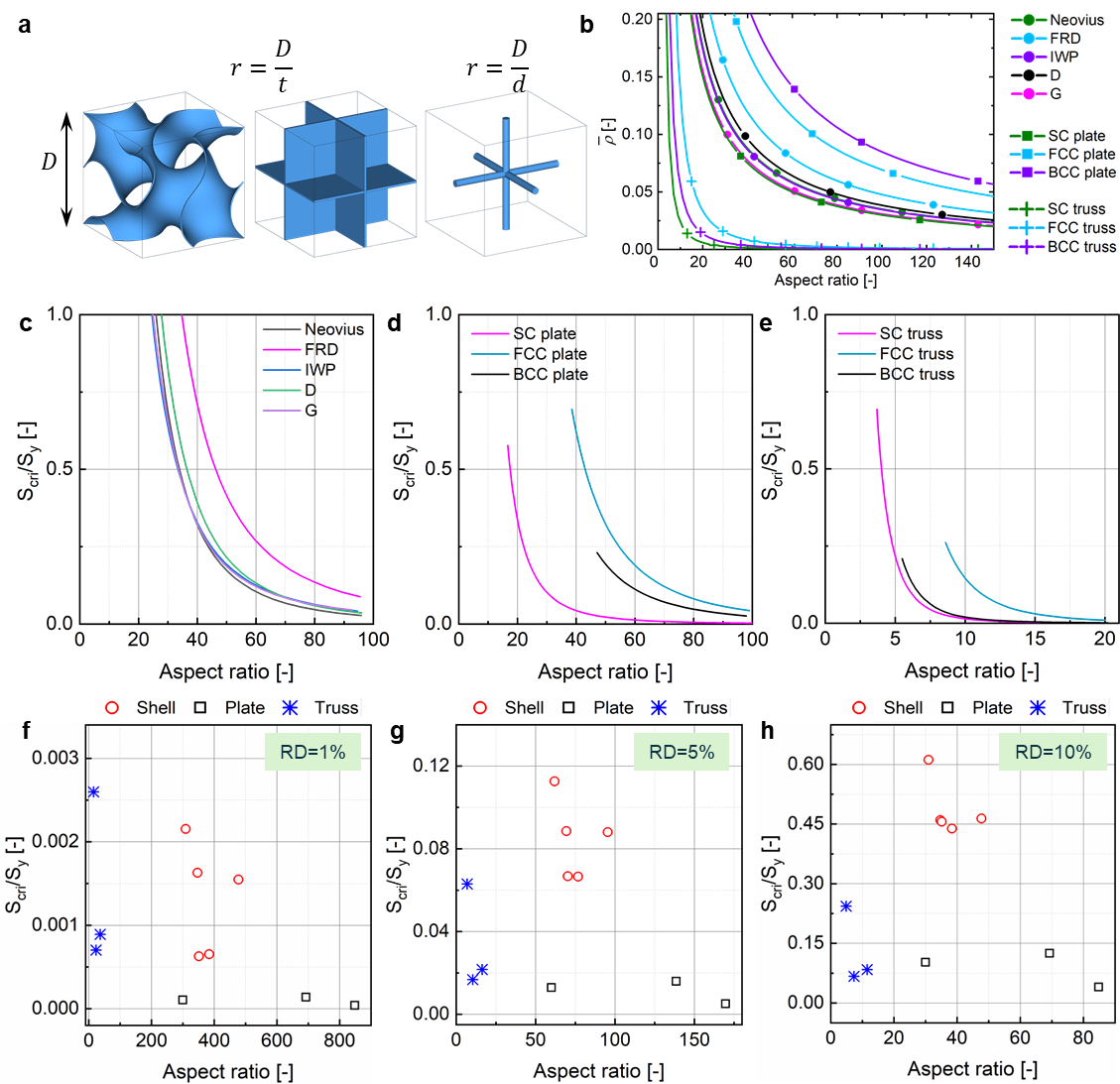


**Figure S21.** The definition of the aspect ratio for three classes of lattices, their RD versus aspect ratio plots, and relative critical buckling strength (*S*_cri_/*S_y_*) versus aspect ratio plots. (a) The definition of the aspect ratios of plate, shell, and truss lattices. (b) RD versus aspect ratio plots of the 11 selected types of lattices. The relative critical buckling strength versus aspect ratio plots of (c) shell, (d) plate, and (e) truss lattices, and scatter plots of relative critical buckling strength versus aspect ratio for the plate, shell, and truss lattices with (f) 1.0%, (g) 5.0%, and (h) 10.0% RDs.

**Supplementary References**

[S1] Y. Kiang, R. Lang, *Appl. Opt*. **1983**, 22, 1296.

[S2] M. Benedetti, A. Du Plessis, R. Ritchie, M. Dallago, S. M. J. Razavi, F. Berto, *Mater. Sci. Eng.: R: Rep.* **2021**, 144, 100606.

[S3] K. A. Brakke, *Exp. Math.* **1992**, 1, 141.

[S4] T. Voisin, J.-B. Forien, A. Perron, S. Aubry, N. Bertin, A. Samanta, A. Baker, Y. M. Wang, *Acta Mater*. **2021**, 203, 116476.

[S5] L. Zhang, J. Lifton, Z. Hu, R. Hong, S. Feih, Addit. Manuf. **2022**, 58, 103038.

**Supplementary Movies**

**Movie S1:** this movie shows the buckling behavior of original and corrugated plate lattice under small strain within 0.01.

**Movie S2:** this movie shows the collapse behavior of original and corrugated plate lattice under large strain within 0.75.
